# Complete plastome sequencing resolves taxonomic relationships among species of *Calligonum* L. (Polygonaceae) in China

**DOI:** 10.1186/s12870-020-02466-5

**Published:** 2020-06-08

**Authors:** Feng Song, Ting Li, Kevin S. Burgess, Ying Feng, Xue-Jun Ge

**Affiliations:** 1 University of Chinese Academy of Sciences, Beijing, 100049 China; 2 State Key Laboratory of Desert and Oasis Ecology, Xinjiang Institute of Ecology and Geography, Chinese Academy of Sciences, Urumqi, 83011 China; 3 Key Laboratory of Plant Resources Conservation and Sustainable Utilization, South China Botanical Garden, Chinese Academy of Sciences, Guangzhou, 510650 China; 4 Department of Biology, Columbus State University, University System of Georgia, Columbus, GA 31907-5645 USA; 5 The Specimen Museum of Xinjiang Institute of Ecology and Geography, Chinese Academy of Sciences, Urumqi, 83011 China

**Keywords:** *Calligonum*, DNA barcodes, Plastid genome, Species resolution

## Abstract

**Background:**

*Calligonum* (Polygonaceae) is distributed from southern Europe through northern Africa to central Asia, and is typically found in arid, desert regions. Previous studies have revealed that standard DNA barcodes fail to discriminate *Calligonum* species. In this study, the complete plastid genomes (plastome) for 32 accessions of 21 *Calligonum* species is sequenced to not only generate the first complete plastome sequence for the genus *Calligonum* but to also 1) Assess the ability of the complete plastome sequence to discern species within the group, and 2) screen the plastome sequence for a cost-effective DNA barcode that can be used in future studies to resolve taxonomic relationships within the group.

**Results:**

The whole plastomes of *Calligonum* species possess a typical quadripartite structure. The size of the *Calligonum* plastome is approximately 161 kilobase pairs (kbp), and encodes 113 genes, including 79 protein-coding genes, 30 tRNA genes, and four rRNA genes. Based on ML phylogenetic tree analyses, the complete plastome has higher species identification (78%) than combinations of standard DNA barcodes (*rbcL* + *matK* + nrITS, 56%). Five newly screened gene regions (*ndhF*, *trnS-G*, *trnC-petN*, *ndhF-rpl32*, *rpl32-trnL*) had high species resolution, where *ndhF* and *trnS*-*G* were able to distinguish the highest proportion of *Calligonum* species (56%).

**Conclusions:**

The entire plastid genome was the most effective barcode for the genus *Calligonum*, although other gene regions showed great potential as taxon-specific barcodes for species identification in *Calligonum*.

## Background

*Calligonum* L. (Calligoneae, Polygonaceae) are xerophytic shrubs distributed in Asia, Northern Africa, and southeastern Europe, although central Asia is the species diversification center for the genus. Many *Calligonum* species are the dominant species in desert vegetation, where they typically have reduced (or absent) leaves and the young branches are the chief organs for photosynthesis [1]. Due to the extreme simplification of vegetative organs, species identification of the four sections in this genus is mostly based on fruit (achene) morphology; *Calliphysa* Borszcz., *Calligonum*, *Pterococcus* Borszcz., and *Medusa* Sosk. et Alexandr. are all typically characterized as having fruit that are membranous or saccate, with narrow wings or bristles at the margins, respectively [2–5]. Nevertheless, the fruit morphology can also be highly variable, making delimitation of species within the genus *Calligonum* troublesome [2]. The estimated number of species varies depending on the treatment: 28–80 species [6]; 174 species reduced to 28 [5]; and 35 species [3].

To help with species identification, a number of molecular analyses have been implemented with little success in *Calligonum*. Although gene regions of the plastid genome (*matK*, *rbcL*, *trnH-psbA*), as well as the nuclear ribosomal internal transcribed spacer (nrITS) region, have been widely used as standard DNA barcodes for species identification in general [7–9], DNA barcoding analyses based on these standard regions, as well as other plastid DNA sequences (*atpB-rbcL*, *trnL-trnF*, *psbK-psbI*) fail to discriminate *Calligonum* species [10–12]. Furthermore, recent molecular sequence analysis [13] has treated five species (*C. mongolicum*, *C. pumilum*, *C. chinense*, *C. alashanicum*, and *C. zaidamense*) as a complex group, *C. mongolicum*. Given such discrepancies, more discerning genetic markers for the genus *Calligonum* are required to solve taxonomic confusion within the group.

The generation and utilization of a complete plastome sequence may be a possible solution to resolve taxonomic relationships in the genus *Calligonum*. Recently, complete plastid genomes have been suggested as a “super-barcode” to overcome the inherent limitations associated with traditional DNA barcoding [14–16]. A genetic sequence of the complete plastome can be easily obtained through a genome skimming approach of high-copy genomic targets, where its conserved gene content, organization and, structure makes it easy to assemble and annotate [17]. Notably, the compete plastome, in addition to all the standard plastid barcodes, should provide a wealth of informative and variable sites for the genetic identification and phylogenetic analyses of plant species [18, 19]: also see e.g., *Ficus* [20], *Lilium* [21], *Panax* [22], *Stipa* [23], *Taxus* [24], and *Diospyros* [25].

Once sequenced, the complete plastome sequence can be screened for potential taxon-specific, hyper-variable gene regions that are likely to be a more cost-effective, yet useful, species identification tool, than the entire plastome [15, 26]. Although this strategy has worked for a number of gene regions across a range of taxa (i.e., the *ycf1* gene region within *Pterocarpus* [27] and *Prunus* [28]; the *trnC*-*rps16*, *trnS*-*trnG*, and *trnE*-*trnM* gene regions for *Panax* [22]; and *trnQ*-*psbK*, *trnR*-*atpA*, *trnS*-*psbZ* and *rpl33*-*rps18* for *Oresitrophe* [26]) to date, there are no reported sequences for the plastomes of any *Calligonum* species, nor has a genome-wide search for taxon-specific barcodes been completed for the group.

To test the power and efficiency of plastome sequences to resolve taxonomic relationships within the genus *Calligonum*, we selected 32 accessions, representing 21 taxa of *Calligonum*, for genome skimming. We addressed the following three objectives: 1) Generate the complete plastome sequence for the genus *Calligonum*; 2) Assess the ability of the complete plastome sequence to discern species within the group, and 3) Screen the plastome sequence for a cost-effective barcode that can be used in future studies to resolve taxonomic relationships within the group.

## Results

### Plastome analysis

**Fig. 1:**
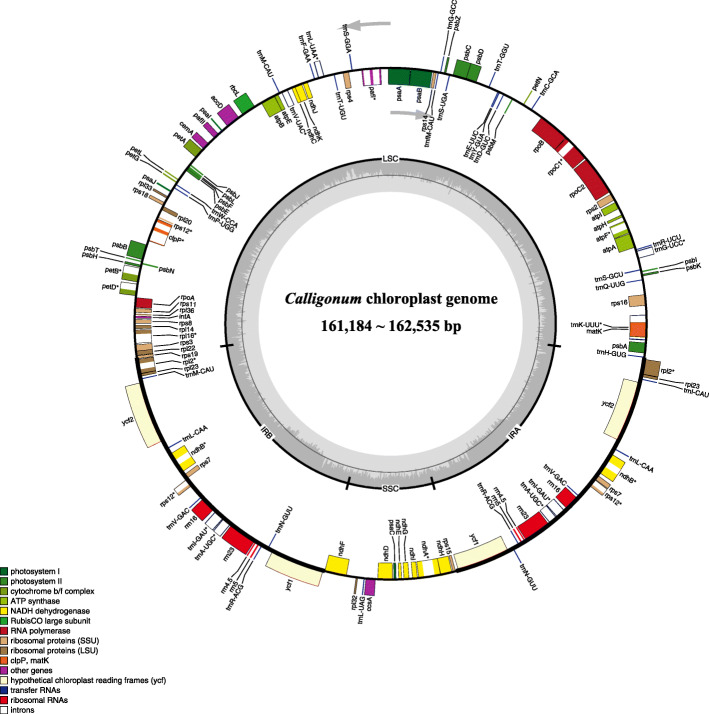
Gene map of the complete chloroplast genome of *Calligonum*. Arrows indicate the direction of transcription: genes located outside the outer circle are transcribed in the counter-clockwise direction, those inside are transcribed in the clockwise direction. Color codes represent different functional gene groups. Inside the middle circle, GC and AT content variations are indicated by darker and lighter gray, respectively

Complete plastomes from 32 accessions of *Calligonum* were submitted to GenBank (Table 1). Plastome size ranged from 161,184 bp (*C. rubicundum*) to 162,535 bp (*C. jeminaicum*). The *Calligonum* plastomes were highly conserved in organization and structure. They showed a typical quadripartite genome organization, including a LSC (Large Single Copy) region (86,766–88,160 bp) and a SSC (Small Single Copy) region (13,286–13,416 bp), which were separated by two IR (Inverted Repeat) regions (30,468–30,552 bp) (Table 1, Fig. 1). The total GC content was 37.50% in the plastomes of *Calligonum* (Table 1), whereas the GC content was higher in the IR region (41.30%) than in the LSC (35.60–35.70%), and SSC (32.40–32.70%) regions.

**Table 1:** Characteristics of *Calligonum* plastomes

sections	Species	Accession ID	Collector	Herbarium /Voucher No.	Total length (bp)	LSC length (bp)	IR length (bp)	SSC length (bp)	GC content	No. of protein-coding genes	No. of tRNA	No. of rRNA	GenBank Accession (plastid)	GenBank Accession (nrITS)
***Pterococcus***	*C. aphyllum*	HM1956	Xue-Jun Ge	IBSC/TLF-120	161,261	86,862	30,526	13,347	37.50%	79	30	4	MN202596	MN327055
	*C. aphyllum*	SS-WY	Ying Feng	XJBI/YDZ-10	161,251	86,853	30,526	13,346	37.50%	79	30	4	MN202595	MN327056
	*C. leucocladum*	HM1945	Xue-Jun Ge	IBSC/TLF-109	161,279	86,836	30,541	13,361	37.50%	79	30	4	MN218643	MN327076
	*C. rubicundum*	HM1954	Xue-Jun Ge	IBSC/TLF-118	161,184	86,766	30,531	13,356	37.50%	79	30	4	MN218648	MN327081
	*C. rubicundum*	SS-HP	Ying Feng	XJBI/FBE-10	161,396	86,974	30,530	13,362	37.50%	79	30	4	MN218649	MN327082
***Medusa***	*C. arborescens*	HM1944	Xue-Jun Ge	IBSC/TLF-108	162,004	87,629	30,526	13,323	37.50%	79	30	4	MN202599	MN327057
	*C. caput-medusae*	HM1955	Xue-Jun Ge	IBSC/TLF-119	162,019	87,632	30,521	13,345	37.50%	79	30	4	MN202600	MN327058
	*C. caput-medusae*	SS-S	Ying Feng	XJBI/YTZ-2	162,043	87,636	30,521	13,365	37.50%	79	30	4	MN202601	MN327059
	*C. ebinuricum*	SS-A1	Ying Feng	XJBI/YAB-1	161,265	86,822	30,541	13,361	37.50%	79	30	4	MN202605	MN327063
	*C. ebinuricum*	SS-A2	Ying Feng	XJBI/SS-A2	161,364	86,946	30,530	13,358	37.50%	79	30	4	MN202606	MN327064
	*C. ebinuricum*	SS-S2	Ying Feng	XJBI/09633	161,290	86,835	30,547	13,361	37.50%	79	30	4	MN202607	MN327065
	*C. gobicum*	SS-G	Ying Feng	XJBI/FXT-6	161,375	86,915	30,552	13,356	37.50%	79	30	4	MN202598	MN327066
	*C. jeminaicum*	SS-J1	Wei Shi	XJBI/SJK-1	162,535	88,160	30,528	13,319	37.50%	79	30	4	MN202608	MN327067
	*C. jeminaicum*	SS-J2	Ying Feng	XJBI/049796	162,459	88,107	30,468	13,416	37.50%	79	30	4	MK854997	MN327068
	*C. juochiangense*	SS-R2	Yan Li	XJBI/CL68	161,339	86,952	30,516	13,355	37.50%	79	30	4	MN202597	MN327072
	*C. korlaense*	SS-K1	Yan Li	XJBI/CL9	161,316	86,914	30,525	13,352	37.50%	79	30	4	MN202612	MN327074
	*C. korlaense*	SS-K2	Ying Feng	XJBI/50852	161,355	86,941	30,531	13,352	37.50%	79	30	4	MN202613	MN327075
	*C. mongolicum*	HM1766	Xue-Jun Ge	IBSC/Q-202	161,316	86,892	30,526	13,372	37.50%	79	30	4	MN218644	MN327077
	*C. pumilum*	SS-XS1	Yan Li	XJBI/YWN-1	161,324	86,904	30,532	13,356	37.50%	79	30	4	MN218645	MN327078
	*C. roborowskii*	SS-TL	Yan Li	XJBI/C58–11	161,367	86,928	30,540	13,359	37.50%	79	30	4	MN218646	MN327079
	*C. roborowskii*	SS-TL2	Yan Li	XJBI/C21	161,329	86,872	30,533	13,391	37.50%	79	30	4	MN218647	MN327080
	*C. taklimakanense*	SS-TK2	Yan Li	XJBI/C61	161,629	87,161	30,541	13,386	37.50%	79	30	4	MN218651	MN327084
	*C. yengisaricum*	SS-Y	Yan Li	XJBI/CL53	161,391	86,975	30,527	13,362	37.50%	79	30	4	MN218652	MN327085
	*C. yengisaricum*	SS-Y2	Ying Feng	XJBI/09650	161,417	86,973	30,541	13,362	37.50%	79	30	4	MN218653	MN327086
***Calliphysa***	*C. junceum*	HM0299	Xue-Jun Ge	IBSC/Ge130299	162,124	87,750	30,526	13,322	37.50%	79	30	4	MK854996	MN327069
	*C. junceum*	HM1946	Xue-Jun Ge	IBSC/TLF-110	162,036	87,650	30,550	13,286	37.50%	79	30	4	MN202609	MN327070
	*C. junceum*	SS-BP	Ying Feng	XJBI/FMD-9	162,081	87,707	30,526	13,322	37.50%	79	30	4	MN202610	MN327071
***Calligonum***	*C. colubrinum*	SS-H	Ying Feng	XJBI/YDG-1	161,321	86,901	30,531	13,358	37.50%	79	30	4	MN202602	MN327060
	*C. cordatum*	SS-X2	Ying Feng	XJBI/YXX-3	161,264	86,861	30,526	13,351	37.50%	79	30	4	MN202603	MN327061
	*C. densum*	SS-MC	Ying Feng	XJBI/YMC-3	161,276	86,873	30,526	13,351	37.50%	79	30	4	MN202604	MN327062
	*C. klementzii*	SS-Q	Ying Feng	XJBI/JQT-7	161,405	86,969	30,526	13,384	37.50%	79	30	4	MN202611	MN327073
	*C. squarrosum*	SS-C	Ying Feng	XJBI/YJFZ-1	161,320	86,900	30,531	13,358	37.50%	79	30	4	MN218650	MN327083

All plastomes encoded 113 unigenes, including 79 protein-coding genes, 30 tRNA genes, and four rRNA genes with identical gene order (Table 1, Fig. 1). None of the regions were inferred to be pseudogenes (Additional file 1: Table S1). Among these genes, five complete protein-coding genes (*rpl2*, *rpl23*, *ycf2*, *ndhB*, *rps7*, *ycf1*); three partial protein-coding genes (*rps19*, *rps12*, *ndhF*), seven tRNA genes (*trnM*^*CAU*^, *trnL*^*CAA*^, *trnV*^*GAC*^, *trnI*^*GAU*^, *trnA*^*UGC*^, *trnR*^*ACG*^, *trnN*^*GUU*^) and four rRNA genes (*rrn16*, *rrn2*3, *rrn4.5*, *rrn5*) were duplicated in the IR regions (Fig. 1).

**Fig. 2:**
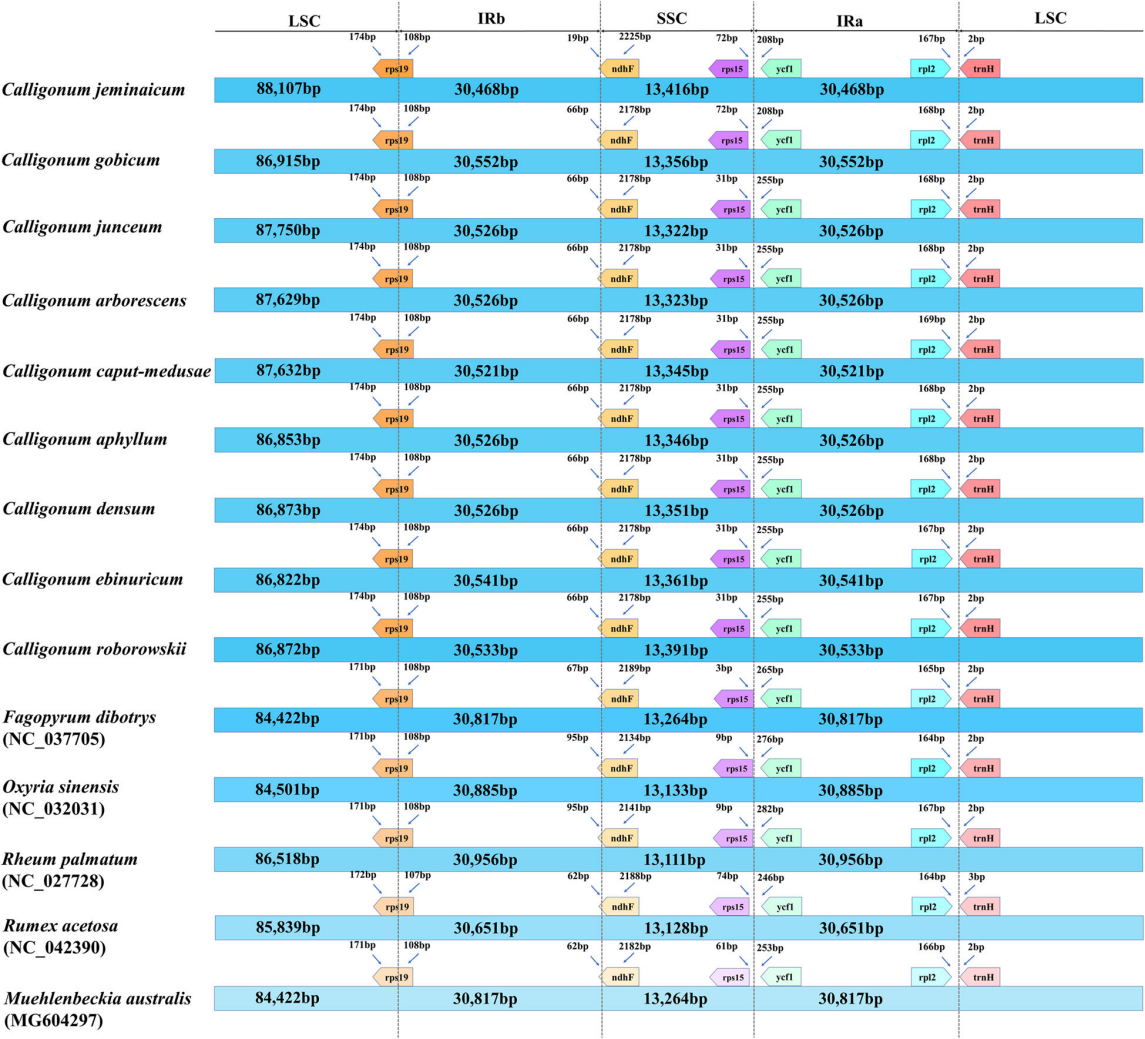
Comparison of the LSC, IR and SSC borders of *Calligonum* and other five Polygonaceae genera, with the *Calligonum jeminaicum* plastome as a reference

Using *C. jeminaicum* as the reference, the homology of 21 *Calligonum* species was investigated to determine the level of sequence divergence (Additional file 2: Figure S1). The complete plastome alignment for the 21 *Calligonum* species showed that there were no rearrangement events among *Calligonum* species (Additional file 5: Figure S3). The plastome sequences were highly similar within the genus *Calligonum*. The LSC/IRb and IRb/SSC borders in the *Calligonum* plastome were positioned within the coding region of *rps19* (with 107–108 bp located at IRb) and *ndhF* (with 19–95 bp located at IRb) genes, respectively (Fig. 2). The intergenic *rps15*-*ycf1* was located in the border of SSC/IRa, whereas the intergenic *rpl2*-*trnH*^*GUG*^ was located in the border of IRa/LSC in *Calligonum* (Fig. 2). There was a slight variation in genome size and IR expansion / contraction (Fig. 2, Additional file 2: Figure S1). Observed plastome length variation was caused by two inserts in *C. jeminaicum* (Additional file 2: Figure S1), which were located in the LSC; one (segment I: about 800 bp) in the intergenic region *rps16*-*trnQ*^*UUG*^, and another (segment II: about 400 bp) in the intergenic region *petN*-*psbM* (see details Additional file 2: Figure S1 and Additional file 4: Table S2).

To estimate selection pressure, the rate of nonsynonymous (*dN*) and synonymous (*dS*) substitutions, as well as the *dN* / *dS* (*ω*) ratio, was determined for 79 protein-coding genes (Additional file 7: Figure S4). In most genes, *dS* were higher than *dN*. The *dN* and *dS* values were 0 to 0.17, and 0 to 0.63, respectively. Most genes showed *ω* ratios less than 0.5, and four genes (*psbI*, *petN*, *psbE*, and *psbL*) had the lowest (close to 0) *ω* ratios (Additional file 7: Figure S4). The *ω* ratios of *rpl23*, *ycf1*, and *ycf2*, ranged from 0.5 to 1.

### Whole plastome for discriminating *Calligonum*

A total 1151 polymorphic sites (0.86%) were detected in the 133,980 bp matrix of 32 *Calligonum* accessions (Table 2). Sequence divergences among 32 *Calligonum* plastomes were compared using nucleotide differences and sequence distances. At the interspecific level, the greatest differentiation occurred between *C. taklimakanense* and *C. jeminaicum* (p-distance = 3.69 × 10^− 3^, different sites = 2867), whereas the closest species were *C. colubrinum* and *C. squarrosum* (p-distance = 0, nucleotide differences is 1) (Additional file 6). At the intraspecific level, the p-distances ranged from 0.2 × 10^− 4^ (*C. aphyllum*) to 8.5 × 10^− 4^ (*C. roborowskii*), and the number of different sites ranged from 14 (*C. aphyllum*) to 388 (*C. roborowskii*) (Additional file 6).

**Table 2:** Discrimination ability of standard and species-specific barcodes and their combinations

DNA barcodes type			length (bp)	Polymorphic sites	Polymorphic sites (%)	Identified species (Success rate)
**Standard DNA barcodes**	nrITS	ITS1 + 5.8S	449	6	1.34	0 (0%)
		5.8S + ITS2	467	16	3.43	1 (11%)
		nrITS	768	22	2.92	1 (11%)
	plastid	*matK*	1527	14	0.92	2 (22%)
		*rbcL*	1428	8	0.56	2 (22%)
		*trnH-psbA*	386	23	5.96	2 (22%)
	combination	*matK + rbcL*	2955	22	0.74	3 (33%)
		*trnH-psbA + matK* + *rbcL*	3341	44	1.32	3 (33%)
		*matK + rbcL +* nrITS	3723	44	1.18	5 (56%)
		*trnH-psbA + matK* + *rbcL* + nrITS	4109	66	1.61	4 (44%)
**Genomic element**	*trnE-T*		995	21	2.11	1 (11%)
	*trnT-L*		1026	36	3.51	2 (22%)
	potential specific-barcodes	*ndhF*	2244	38	1.69	5 (56%)
		*ndhF*-*rpl32*	993	28	2.82	4 (44%)
		*rpl32*-*trnL*	632	16	2.53	4 (44%)
		*trnC*-*petN*	900	82	9.11	4 (44%)
		*trnS-G*	1233	26	2.11	5 (56%)
		Combination*	5795	200	3.45	6 (67%)
	Complete plastid genomes (only one IR)		133,980	1151	0.86	7 (78%)

**Fig. 3:**
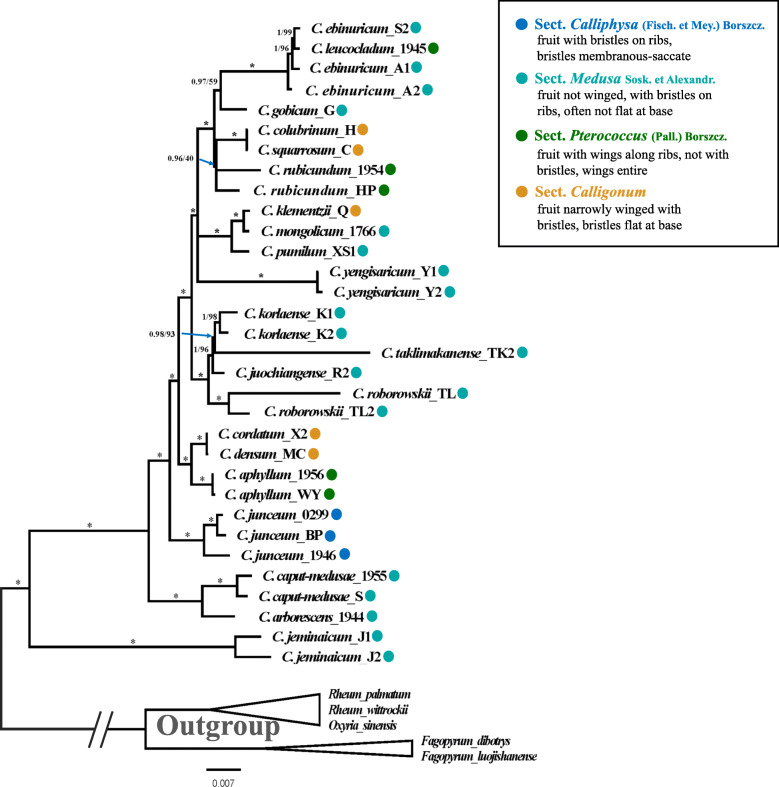
Phylogenetic relationships of 32 *Calligonum* accessions inferred from ML and BI tree. Numbers above branches indicate posterior probabilities (PP, left) and the ML bootstrap values (BS, right)**.** Branches with* have PP = 1 and BS = 100%. The sections follow the system of Soskov (2011). The colors represent different sections

Based on the plastomic matrix, identical ML and BI trees were obtained (Fig. 3). The monophyly of the genus *Calligonum* was strongly supported in both cases. The infrageneric phylogeny was well resolved and most nodes were strongly supported (Fig. 3). Only two nodes, one that includes *C. colubrinum*, *C. squarrosum* and *C. rubicundum* (BS = 40%, PP = 0.96), and another that includes *C. ebinuricum*, *C. leucocladum*, and *C. gobicum* (BS = 59%, PP = 0.97), were not well supported. The discriminatory power of the plastomes was assessed by investigating the monophyly, and branch support recovered in those species where multiple accessions were sampled. Seven of the nine species (78%) that had more than one accession were resolved as reciprocally monophyletic except for *C. ebinuricum* and *C. rubicundum* (Fig. 3). The relationship among samples that had one accession was well supported (BS > 93%, PP > 0.98), only *C. gobicum* (BS = 59%, PP = 0.97) was the exception (Fig. 3).

The phylogenetic tree did not support the division of three or four sections in *Calligonum* [5, 29]. Only sect. *Calliphysa*, containing one species (*C. junceum*), was well supported (BS = 100%, PP = 1.00). Species from the other sections often formed one clade. For example, *C. aphyllum* from sect. *Pterococcus* formed one well supported (BS = 100%, PP = 1.00) clade with *C. densum* and *C. cordatum,* both of which are from sect. *Calligonum*.

### Analyses of potential barcodes

Due to the PCR failure for ITS [12], we de novo assembled nrITS from genome skimming data, which included the ITS1, 5.8S, and ITS2 regions. Alignments and concatenation of 32 nrITS sequences yielded a 768 bp matrix in length, including 22 polymorphic sites (2.92%) (Table 2). The discriminatory power analysis based on the BI method exhibited weak resolution at most nodes. For nine species with multiple accessions, only *C. ebinuricum* was recovered as a supported monophyletic clade (PP = 0.86, Fig. 5a), with an 11% success rate. ITS2 (15 polymorphic sites) harbors more variability than ITS1 (5 polymorphic sites), and revealed higher discrimination power (Table 2).

For the three standard plastid barcodes, complete *matK*, *rbcL* and *trnH-psbA* sequences had the same resolution power (22%). However, the combinations of *matK + rbcL* and that of *trnH-psbA + matK* + *rbcL* slightly increased identification power to 33% (Table 2, Additional file 9: Figure S6C-G). When the plastid barcodes were combined with nrITS, the identification rate increased to 44 (*trnH-psbA + matK + rbcL* + nrITS) and 56% (*matK + rbcL* + nrITS) (Table 2, Fig. 5b, Additional file 9: Figure S6H). Both combinations generated tree topologies that were similar to the complete plastome data sets, although their resolution power was lower than that of the plastid genomes (Table 2).

**Fig. 4:**
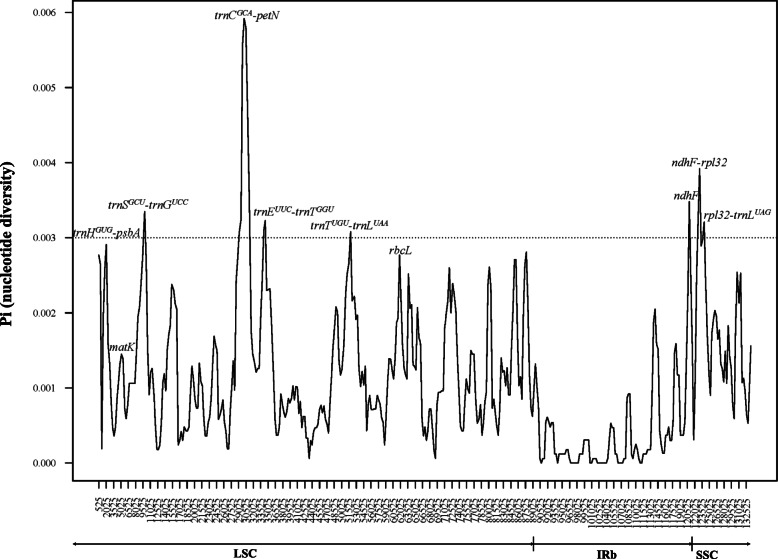
Sliding window analysis of the entire chloroplast genome of *Calligonum* species (window length: 1000 bp; step size: 300 bp). X-axis: position of the window; Y-axis: nucleotide diversity of each window

**Fig. 5:**
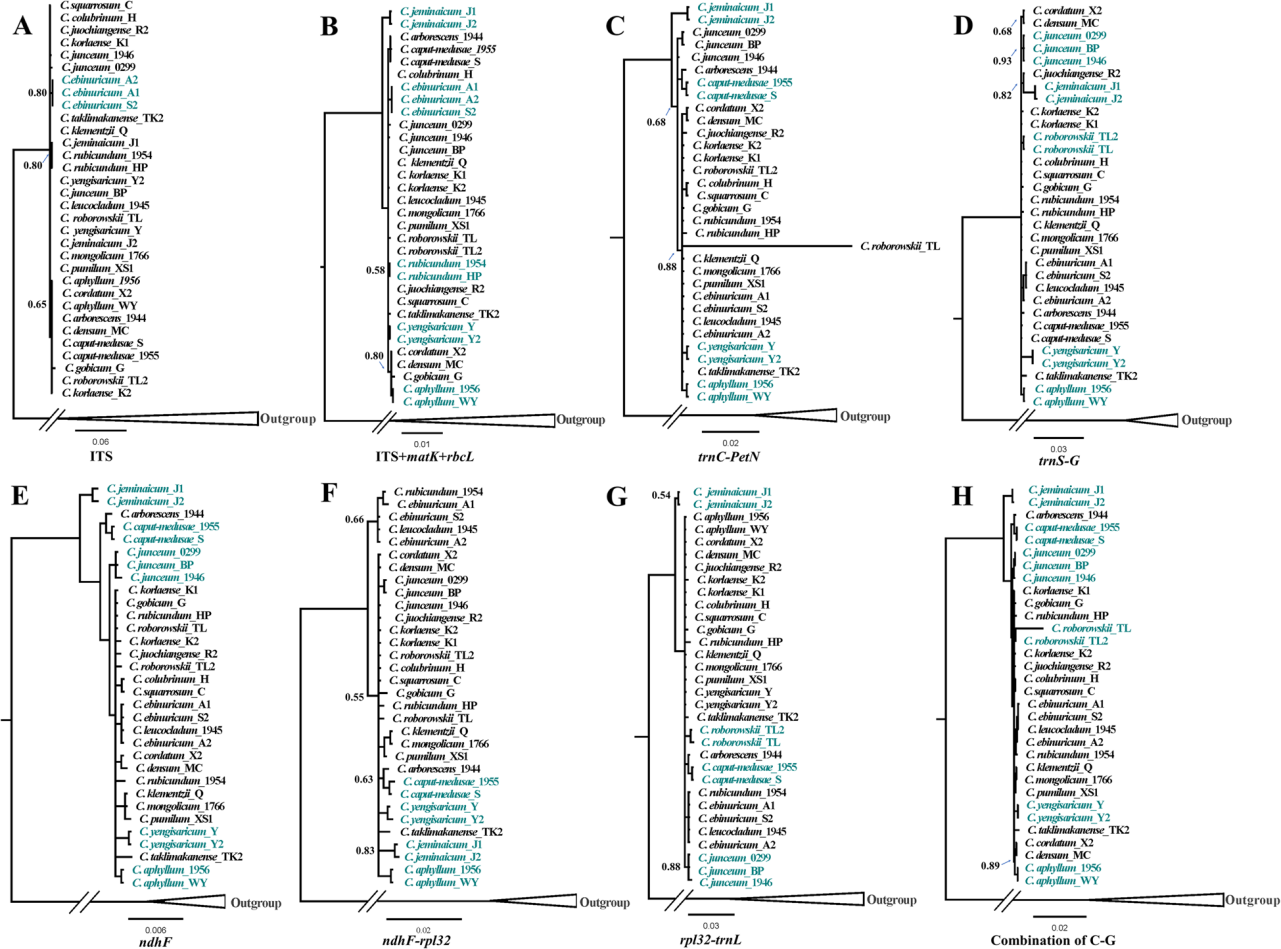
Bayesian tree inferred from two types of barcodes. a: ITS; b: ITS+*matK* + *rbcL* (standard DNA barcodes); c: *trnC*-*petN*; d: *trnS-G*; e: *ndhF*; f: *ndhF*-*rpl32*; g: *rpl32*-*trnL*; h: combination of potential specific-barcodes. The colors represent the species were reciprocally monophyletic. Number on the tree are posterior probabilities of nodes (values > 0.95 not shown)

In this study, the variability of additional, potential plastid regions was quantified with nucleotide diversity (Pi), which was calculated with a sliding window (window length = 1000 bp and step size = 300 bp). The values of nucleotide diversity (Pi) ranged from 0 to 0.0059. Seven hyper-variable regions (Pi > 0.003) in these genomes were identified, six of which are intergenic regions (i.e., *trnS-G*, *trnC-petN*, *trnE-T*, *trnT-L*, *ndhF-rpl32*, and *rpl32-trnL*). Only one protein-coding region (*ndhF*, Fig. 4) showed high nucleotide diversity within *Calligonum*. These hyper-variable regions were all located in the LSC and SSC regions (Fig. 4). The polymorphic site number in these seven regions was remarkably higher than that in standard DNA barcodes (*rbcL*, *matK, trnH*-*psbA*, nrITS) (Table 2). Their power as potential taxon-specific barcodes was tested through a tree-based method. The species discrimination rates (range from 44 to 56%, Table 2, Fig. 5) were much higher than that of *rbcL* and *matK*, except the *trnT-L* (discrimination rate of 22%, Table 2) and *trnE-T* regions (discrimination rate of 11%, Table 2). Among these five regions, *ndhF* and *trnS-G* had the highest discrimination rate (56%) (Table 2, Fig. 5d-e). The combination of the five gene regions (*ndhF*, *trnS-G*, *trnC-petN*, *ndhF-rpl32*, *rpl32-trnL*) increased the identification of species to 67% (Table 2, Fig. 5h).

## Discussion

### Plastome features

In this study, we generated 32 complete *Calligonum* plastomes. The plastomes in *Calligonum* are highly conserved and ranged in size of 161,184 to 162,535 bp. When compared to the plastomes of the other Polygonaceae genera (e.g., *Fagopyrum* [30], *Rumex* [31], *Oxyria* [32]), all the plastomes generated in this study exhibited typical plastome structure, gene order and content (Fig. 1). In addition, the GC content of *Calligonum* (37.50%) was similar to that of *Fagopyrum* (37.80–38.0%) [30], *Rumex acetosa* (37.20%) [31], and equal to that of *Oxyria sinensis* (37.50%) [32]. Inverted repeat (IR) contraction and expansion is a common evolutionary phenomenon and may cause variation in plastome length [33]. Nonetheless, the IR regions of the *Calligonum* plastomes varied slightly from 30,468 bp to 30,552 bp (Fig. 2). Compared to other Polygonaceae genera that have plastome data in GenBank, the IR region in *Calligonum* is more conserved than the Large Single Copy (LSC) and Small Single Copy (SSC) regions, where most differences were observed in the intergenic and intron regions (Additional file 3: Figure S2). One of the two inserts (segment I) found in *C. jeminaicum* also existed in *Muehlenbeckia australis*, *Oxyria sinensis*, and *Rheum palmatum*, whereas it was absent in *Fagopyrum*, *Rumex* and *Calligonum* (except *C. jeminaicum*, *C. junceum*, *C. arborescens*, and *C. caput-medusae,* Additional files 2, 3: Figure S1, S2). The other insert (segment II) was only absent in *Calligonum* (except *C. jeminaicum*). Collectively, these results indicate that intergenic and intron variation are a significant source of length variation in *Calligonum*, compared to other genera in the Polygonaceae (Fig. 2, Additional file 3: Figure S2).

### Taxonomic resolution based on the complete plastome

Complete plastomes have been suggested as having the potential to increase species resolution among plant species [18, 19], and have been used to discriminate species in a number of taxa that are difficult to resolve (e.g., *Ficus* [20]; *Panax* [22]; *Taxus* [24]; *Diospyros* [25]). In our study, seven of the nine species (78%, Table 2) in *Calligonum* that have more than one accession, were correctly identified to species. Among the seven species, *C. roborowskii* revealed the highest intraspecific variation (388 variable sites), where two individuals showed obvious branch length difference (Fig. 3). Previous studies have revealed high genetic variation among populations of *C. roborowskii* (AMOVA: 91.19%, *Gst*: 0.818) that also have significant phylogeographical structure based on cpDNA data [34]. In our study, we also found that those species with a single accession were well resolved with strongly supported nodes in our phylogenic tree (Fig. 3). The wide distribution range, patchiness of populations and short-distance seed dispersal due to gravity, all likely contribute to genetic differentiation in *C. roborowskii* [34]. Collectively, these results indicate that the complete plastome sequence is an effective tool for species discrimination in *Calligonum* and are in-line with current taxonomic treatments. For example, in the Flora of China [3], *C. juochiangense* was reduced as a synonym of *C. pumilum*, however, based on further morphological analysis, Feng et al. [35] found that both species are quite different from each other and that they should be considered as two independent species. Based on our plastome phylogeny (Fig. 3), *C. juochiangense* formed one clade with *C. korlaense* and *C. taklimakanense* with strong support, and separate from *C. pumilum*. Our plastome results support their entities as separate species taxonomy. Although *C. colubrinum* and *C. squarrosum* were treated as different species in the Flora of China [3], they have very similar morphological characters, but differ in fruit size, color and location of bristles on achenes. However, these characters may change at the different development stages, and there is no discontinuous variation between these two species. There is a single nucleotide site difference between the plastome of *C. colubrinum* and *C. squarrosum*, which suggests they are indeed the same species and *C. squarrosum* N. Pavlov (1933) should be treated as a synonym of *C. colubrinum* E. Borszcow (1860).

Although our sampling only covered 21 species in *Calligonum,* these species represented all the sections in the classifications of *Calligonum* [5](Fig. 3, Additional file 8: Figure S5), with the exception of the species from North Africa and East Mediterranean due to the sampling difficulty. The plastome data presented in this study provide further delineation of taxa within the group. For example, neither infrageneric classification of the genus *Calligonum* [5, 29] was supported in this study (Fig. 3, Additional file 8: Figure S5). Furthermore, our results are in contrast with the most recent taxonomic treatment of *Calligonum*, Sosk. [5], which delineates 28 species and many of which have been reduced to synonyms: *C. gobicum*, *C. korlaense*, *C. yengisaricum*, and *C. roborowskii* have been reduced to the synonym of *C. litwinowii* Drob.; and *C. pumilum* and *C. jeminaicum* to that of *C. rubescens*. Although in our study the polymorphic site ratio is relatively low for the complete plastome (0.86%), the total number of polymorphic sites (1151) is relatively high, indicating that complete plastomes are likely an effective tool for solving taxonomic issues within this group of taxa, especially in genera that have many closely related species (i.e., those that have experienced recent speciation).

Although the *Calligonum* plastome showed relatively high species resolution in this study, approximately 20% of the species could not be successfully identified. *Calligonum* species are known to interbreed in sympatry [13, 34, 36], and it seems likely that in particular, interspecific hybridization may have caused the lack of resolution for *C. ebinuricum* and *C. rubicundum*. For example, three *C. ebinuricum* accessions formed one clade with *C. leucocladum* (BS = 100%, PP = 1.00), however, *C. ebinuricum* alone formed a monophyletic clade, with strong support, in the nrITS phylogeny. Hybridization or introgression has been suggested as the reason for conflicting phylogenic patterns between paternally inherited nuclear genes and maternally inherited plastid genes [37, 38], and thus provides a plausible reason why *C. leucocladum* shares its plastome sequence with *C. ebinuricum*. Similarly, *C. rubicundum* accessions formed a single clade with *C. colubrinum* and *C. squarrosum* (BS = 40%, PP = 0.96), which are of known hybrid origin [5]. The fact that both *C. ebinuricum* and *C. rubicundum* were sampled from cultivated plants at Turpan Eremophytes Botanical Garden not only highlights the possibility that introgression among closely related species in ex situ plant collections is possible [39], but also serves as a caution, that in some cases, utilizing such collections to test species resolution may be a problem.

### Screening the entire plastome for potential DNA barcodes

When screening the complete plastome sequence of *Calligonum* to find suitable barcode regions to identify species in the genus, we first assessed species resolution for a suite of standard DNA barcodes that have been used to assess species resolution in other taxa. In our study, on average, species resolution was low for all the standard DNA barcodes that were screened. In addition, the complete *matK*, *rbcL*, *trnH-psbA* intergenic region, and nrITS sequences were successfully retrieved from the genome skimming data. As a single barcode, species resolution of these gene regions was very low, which ranged from 11% (ITS) to 22% (*rbcL*, *matK*, *trnH*-*psbA*). Their combination slightly increased species resolution from 33% (*rbcL* + *matK*), 33% (*rbcL* + *matK* + *trnH*-*psbA*) to 55% (*rbcL* + *matK* + ITS) (Table 2). These results verified those of previous studies that also showed relatively low resolution rates [12, 36], even though we were able to sequence and screen longer segments (i.e., the complete gene region) of the standard DNA barcodes. There are three possible reasons for the high rates of species identification failure for these DNA barcodes in *Calligonum*: 1) the current taxonomy for the genus is inaccurate; 2) past hybridization events have blurred species boundaries; and 3) recent speciation events have resulted in coalescent failure of the plastid genome [13, 34, 38, 40]. Although the number of recognized species for *Calligonum* varies among monographs [3, 5], the genus is thought to have undergone recent and rapid diversification in the arid deserts of Western Central Asia [41, 42], which may contribute the failure of DNA barcoding to discriminate among *Calligonum* species.

As a biparental inherited marker, nrITS (or ITS2) usually reveals higher species resolution than plastid DNA barcodes [9, 43]. However, nrITS was highly conservative in *Calligonum*, having relatively few polymorphic sites (22, 2.92% of the gene region). As a result, species resolution of nrITS (11%) was even less than the three plastid standard barcodes combined. This result may be due to the young age of this genus [41, 42], frequent hybridization [13] and/or introgression, where most hybridization events in *Calligonum* have been documented between relatively young species that have diverged since the Quaternary [5]. For example, experimental interspecific hybridization among predominantly self-incompatible taxa from sect. *Medusa* showed high fruit sets suggesting no genetically based reproductive barrier [13]. In addition, to these three plausible biological processes, the nrITS consensus sequence in our study was retrieved and assembled based on a seed-and-extend strategy using genome skimming data. This alignment algorithm retrieves the alleles in relatively high frequency, and thus may underestimate the number of polymorphic sites associated with our study species [44] (see Additional file 10, Additional file 11: Table S3). Collectively, these results suggest that nrITS is unable to discern among most *Calligonum* species, and this constraint should be considered in future studies.

### Screening of additional potential barcode regions

DNA barcoding for plants, in general, remains a challenge and, due to the lack of genetic variation for standard barcode gene regions, it is common that closely related, congeneric species share similar barcodes [15, 45–48]. For example, molecular analyses using standard DNA barcodes have failed to differentiate species in *Solanum* sect*. Petota* (wild potatoes) [49], *Salix* [45], *Curcuma* [50], and *Euphrasia* [16], to name a few. Lineage-specific (or taxon-specific) barcodes, however, may enhance species discrimination rates because they typically provide more genetic information within a particular group of species compared to the use of standard DNA barcodes typically used across taxa of broad phylogenetic dispersion. In addition, and compared to complete plastome sequencing, the use of taxon-specific barcode regions are certainly more cost-effective for the large-scale assessment of species-rich genera [15]. In this study, among the new regions that we screened for *Callignoum*, five (*ndhF*, *trnS-G*, *trnC-petN*, *ndhF-rpl32*, *rpl32-trnL*) had species resolution rates that ranged from 44 to 56% (Table 2), which is comparable to results found in *Quercus* [51], *Diospyros* [25], and *Panax* [22]. Among these regions, *ndhF* and *trnS-G* had the highest species discrimination (56%), and in combination (67%) (Table 2), for our study taxa. When considering the cost and time associated with complete plastome sequencing, it is likely that these gene regions have great potential as a *Calligonum*-specific barcode in future studies.

Rapid and cost-effective development of high-throughput sequencing technology has allowed for a rapid increase in the number of complete plastomes available on GenBank (4692 plant species as Feb. 21, 2020; https://www.ncbi.nlm.nih.gov/genome/organelle/). Although complete plastome sequencing is a heavy burden for many laboratories, our contribution to this increasing dataset will make it easier to find taxon-specific barcodes based on plastome data. For those genera lacking plastome data at GenBank, we suggest the sequencing of a few species, at relatively low cost, to establish plastome sequences that can then be screened for taxon-specific barcodes. We suspect that in the future, the plastome will be widely applied as “the plant barcode 2.0” in many related fields [19, 52]. For those genera or species complex with rapid radiation or frequent hybridization, we also suggest that future barcoding studies couple plastome screening with targeted enrichment methods [19, 52] that sample the wealth of genetic resources stored, yet relatively untapped, in the nuclear genome.

## Conclusions

The use of standard DNA barcodes for species identification in *Calligonum* is insufficient. In this study, we tested whole plastomes, standard DNA barcodes and hyper-variable, taxon-specific regions for rates of species resolution in the genus. Among these genetic tools, complete plastomes greatly improved species resolution in *Calligonum* and a number of gene regions showed high potential to be used as taxon-specific barcodes in future studies.

## Methods

### Taxon sampling and DNA sequencing

In total, 32 samples representing 21 species of *Calligonum* [5, 53] were collected from northwestern China (Table 1); only three species in China were not included in this study. No specific permissions were required for the relevant locations/activities. Among the 21 species, nine species had more than one individual sampled. The nomenclature system for this study follows the Flora Reipublicae Popularis Sinicae (FRPS) [54] and the Flora of China (FOC) [3]. Voucher specimens were deposited in the Herbarium of the Xinjiang Institute of Ecology and Geography, Chinese Academy of Sciences (XJBI) and the Herbarium of South China Botanical Garden (IBSC).

Total genomic DNA was extracted from approximately 100 mg of silica-dried branch material. Isolation protocols followed the cetyltrimethyl ammonium bromide (CTAB) method [55]. DNA extracts were fragmented for 300 bp short-insert library construction and sequenced − 2 × 150 bp paired-end (PE) reads on an Illumina HiSeq X-Ten instrument at the Beijing Genomics Institute (BGI, Shenzhen, China). The raw reads were assessed by FastQC 0.11.5 (http://www.bioinformatics.babraham.ac.uk/projects/fastqc/) and edited using Trimmomatic 0.35 [56] to remove adapters and low-quality bases. After removing low quality reads and adaptor sequences, an ~ 3.0 G bp paired-end clean read was obtained for each sample.

### Plastome and nrDNA assembly and annotation

The clean data were assembled using NOVOPlasty v1.1 [57], with a reference genome of *Fagopyrum tataricum* (Polygonaceae) (GenBank accession no. NC_027161). Clean reads were then re-mapped to the preliminary genome and the complete plastid genome sequences were adjusted using Bowtie 2 v2.3.4.1 [58] and SAMtools v1.9 [59]. The finished plastid genomes were annotated with DOGMA [60], and GeSeq [61], then adjusted manually using Geneious v 11.0.2 [62]. Gene start and stop codons were determined by comparison to the genome of *F. tataricum*. Finally, the annotated plastid genomes were submitted to GenBank (Table 1) and Organellar Genome Draw [63] was used to illustrate a circular genome map.

Two steps were adopted to complete nrITS sequence reconstruction. Firstly, the nuclear ribosomal (nr) ITS sequence of *G. junceum* (GenBank accession no. AB542774) was used as the reference to assemble the entire nrITS sequence (ITS1, 5.8S, and ITS2). Sequence assembly followed the same procedures described above. Each assembled sequence served as a reference sequence for the next steps. Secondly, clean reads were mapped to the new obtained reference using Bowtie 2 v2.3.4.1 [58] and SAMtools v1.9 [59], resulting in a BAM file with only mapped reads. The BAM file was then imported into Geneious V. 11.0.2 [62] and consensus sequences were extracted with default settings. Each consensus sequence served as the final nrITS sequence and was annotated by comparison to the reference sequence and then submitted to GenBank (Table 1).

### Variation analyses

To illustrate interspecific sequence variation and gene organization of the entire plastid sequences among each of the 21 species, we used mVISTA software with the LAGAN model [64]. The alignments, with annotations, were visualized using *C. jeminaicum* as a reference, which was generated in the present study. Mauve v1.1.1 (a plugin within Geneious v 11.0.2) [65] was used for alignment and for the detection of gene rearrangements and inversions among *Calligonum* taxa. Sliding window analysis (DnaSP v6 [66]) was conducted to generate Pi values of the plastid genomes. Evolutionary divergence (nucleotide differences and p-distances) among the 32 accessions were evaluated using MEGA X [67]. Hyper-variable regions were defined as a region with relatively high nucleotide diversity (Pi) and high species resolution. The step size was set to 300 bp, with a 1000 bp window length, and regions with the Pi value > 0.003 (more than half of the maximum) were extracted to assess species resolution (see Discriminatory power analysis described below).

To detect whether plastid genes were under selection pressure, the ratio of nonsynonymous (*dN*), synonymous (*dS*) and *ω* (*dN/dS*) values of each protein coding gene in the *Calligonum* plastid genomes were analyzed using CodeML in PAML Version 4.9d [68] with a One-ratio model (model = 0, seqtype = 1, NSsites = 0). Positive selection is detected if the value of *dS*, summed over all branches on the tree, is > 0.5 (PAML FAQ, http://saf.bio.caltech.edu/saf_manuals/pamlFAQs.pdf).

### Discriminatory power analysis

A tree-based method was used to investigate the power and efficiency of plastome sequences for species identification. The discriminatory power was assessed by monophyly and the branch support recovered in those species with multiple accessions. The DNA sequences for the complete plastid genomes (after removing one inverted repeat), and potential DNA barcode regions, were aligned using the default option implemented in MAFFT version 7 [69]. The most appropriate model of nucleotide substitution for each nucleotide sequences was determined by the Akaike Information Criterion (AIC) in jModeltest v 2.1.10 [70]; results are listed in Additional file 12: Table S4. Bayesian inference (BI) was performed using MrBayes 3.2.6 [71] with Markov chain Monte Carlo simulations algorithm (MCMC) for 1 *×* 10^6^ generations with four incrementally-heated chains. Each matrix was given its own optimal model (Additional file 12: Table S4). Maximum likelihood (ML) trees were generated in RAxML 8.2.10 [72] with 1000 replicates. The trees were viewed and edited with FigTree v1.4.3 (http://github.com/rambaut/figtree/). In all analyses, the five Polygonaceae species were chosen as outgroups: *Rheum palmatum* (NC_027728/ AY207370), *R. wittrockii* (NC_035950/ KF258686), *Fagopyrum luojishanense* (NC_037706), *F. tataricum* (NC_027161), and *F. dibotrys* (NC_037705/ JN235080).

## Supplementary information

**Additional file 1: Table S1.** List of genes in the chloroplast genome for 21 *Calligonum* species *Gene with intron (S).

**Additional file 2: Figure S1.** Sequence identity plots for 21 assembled *Calligonum* chloroplast. Genomes, with *C. jeminaicum* as reference (left). Special insertion (or deletion) test results (right). Segments **I**: about 800 bp, segments **II**: about 400 bp.

**Additional file 3: Figure S2.** Sequence identity plots for six Polygonaceae genera plastid genomes, with *C. jeminaicum* as a reference (left). Red boxes represent two special insertion (or deletion) segments **I** (about 800 bp) and **II** (about 400 bp).

**Additional file 4: Table S2.** Primers and samples for special insertion test.

**Additional file 5: Figure S3.** Genome rearrangement events of 21 assembled *Calligonum* species.

**Additional file 6.** Excel spreadsheet of the numbers of nucleotide substitutions and sequence distance in 32 complete cp genomes (Sheet1). The upper triangle shows the number of nucleotide substitutions and the lower triangle indicates the number of sequence distance in complete cp genomes.

**Additional file 7: Figure S4.** The *dN* / *dS* (*ω*) value of protein-coding genes from *Calligonum* plastid genomes.

**Additional file 8: Figure S5.** Three sections of *Calligonum* based on the flora of Iran (Rechinger & Schiman-Czeika, 1986). The colors represent different sections. Numbers above branches indicate posterior probabilities (PP, left) and the ML bootstrap values (BS, right)**.** Branches with* have PP = 1 and BS = 100%.

**Additional file 9: Figure S6.** Bayesian tree inferred from different core-barcodes of the *Calligonum*. Numbers above branches indicate posterior probabilities (**A**: ITS1 + 5.8S; **B**: 5.8S + ITS2; **C**: *matK*; **D**: rbcL **E**: *matK* + *rbcL*; **F**: *trnH-psbA*; **G**: *matK* + *rbcL* + *trnH-psbA*; **H**: *matK* + *rbcL* + *trnH-psbA* + ITS; **I**: *trnE-T*; **J**: *trnT-L*).

**Additional file 10.** Excel spreadsheet of the heterozygous sites and inter-individual polymorphic sites of nrITS sequences. * represents the sequence from Genbank. △ = CGGAGATC.

**Additional file 11: Table S3.** nrITS sequence assembly information.

**Additional file 12: Table S4.** Molecular models selected for all the dataset.

## Data Availability

All complete plastid genome and nrITS sequences used in this study are available from the National Center for Biotechnology Information (NCBI) (see Table 1).

## Abbreviations

AIC: Akaike Information Criterion
BI: Bayesian Inference
BS: Bootstrap
CTAB: Cetyltrimethyl ammonium bromide method
DnaSP: DNA Sequences Polymorphism
DOGMA: Dual Organellar Genome Annotator
IR: Inverted repeat
LSC: Large single copy
MCMC: Markov Chain Monte Carlo
ML: Maximum Likelihood
NCBI: National Center for Biotechnology Information
nrITS: Nuclear ribosomal internal transcribed spacer
rRNA: Ribosomal RNA
SSC: Small single copy
tRNA: Transfer RNA
