# Supplementary material for: Complete plastome sequencing resolves taxonomic relationships among species of Calligonum L. (Polygonaceae) in China
Source: BMC Plant Biol. 2020 Jun 8;20:261. doi: 10.1186/s12870-020-02466-5 (PMC7282103; doi:10.1186/s12870-020-02466-5)
Supplement: Supplementary file 1 — Additional file 1: Table S1. List of genes in the chloroplast genome for 21 Calligonum species *Gene with intron (S). [file 12870_2020_2466_MOESM1_ESM.docx]

**Table S1** List of genes in the chloroplast genome for 21 *Calligonum* species. *Gene with intron (S)

| **Category** | **Gene Group** | **Gene name** |
| --- | --- | --- |
| Self-replication | Ribosomal protein (small subunit) (14) | rps2 rps3 rps4 rps7 (x2) rps8 rps11 *rps12 (x2) rps14 rps15 *rps16 rps18 rps19 |
|  | Ribosomal protein (large subunit) (11) | *rpl2 (x2) rpl14 *rpl16 rpl20 rpl22 rpl23 (x2) rpl32 rpl33 rpl36 |
|  | RNA polymerase (4) | rpoA rpoB *rpoC1 rpoC2 |
|  | Transfer RNAs (37) | trnA-UGC (x2) trnC-GCA trnD-GUC trnE-UUC trnF-GAA trnfM-CAU trnG-GCC *trnG-UCC trnH-GUG trnI-CAU *trnI-GAU (x2) *trnK-UUU trnL-CAA (x2) *trnL-UAA trnL-UAG trnM-CAU(x2) trnN-GUU (x2) trnP-UGG trnQ-UUG trnR-ACG (x2) trnR-UCU trnS-GCU trnS-GGA trnS-UGA trnT-GGU trnT-UGU trnV-GAC (x2) *trnV-UAC trnW-CCA trnY-GUA |
|  | Ribosomal RNAs (8) | rrn4.5 (x2) rrn5 (x2) rrn16(x2) rrn23(x2) |
| Photosynthesis | Photosystem Ⅰ(5) | psaA psaB psaC psaI psaJ |
|  | Photosystem Ⅱ(15) | psbA psbB psbC psbD psbE psbF psbH psbI psbJ psbK psbL psbM psbN psbT psbZ |
|  | Cytochromee b/f complex | petA *petB *petD petG petL petN |
|  | ATP synthase (6) | atpA atpB atpE *atpF atpH atpI |
|  | NADH dehydrogenase (12) | *ndhA *ndhB (x2) ndhC ndhD ndhE ndhF ndhG ndhH ndhI ndhJ ndhK |
|  | Rubisco large subunit (1) | rbcL |
| Other genes | Maturase (1) | matK |
|  | membrane protein (1) | cemA |
|  | Acetyl-CoA carboxylase gene (1) | accD |
|  | ATP-dependent protease subunit (1) | *clpP |
|  | c-type Cytochrome biogenesis (1) | ccsA |
|  | Assembly/stability of photosystemⅠ(2) | * pafI pafII |
|  | Conserved reading frames (ycfs) (4) | ycf1 (x2) ycf2 (x2) |
|  | Translation-related gene (1) | infA |
