# Supplementary figures and images for: Complete plastome sequencing resolves taxonomic relationships among species of Calligonum L. (Polygonaceae) in China
Source: BMC Plant Biol. 2020 Jun 8;20:261. doi: 10.1186/s12870-020-02466-5 (PMC7282103; doi:10.1186/s12870-020-02466-5)

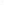 gene  
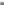 exon  
 UTR  
 CNS

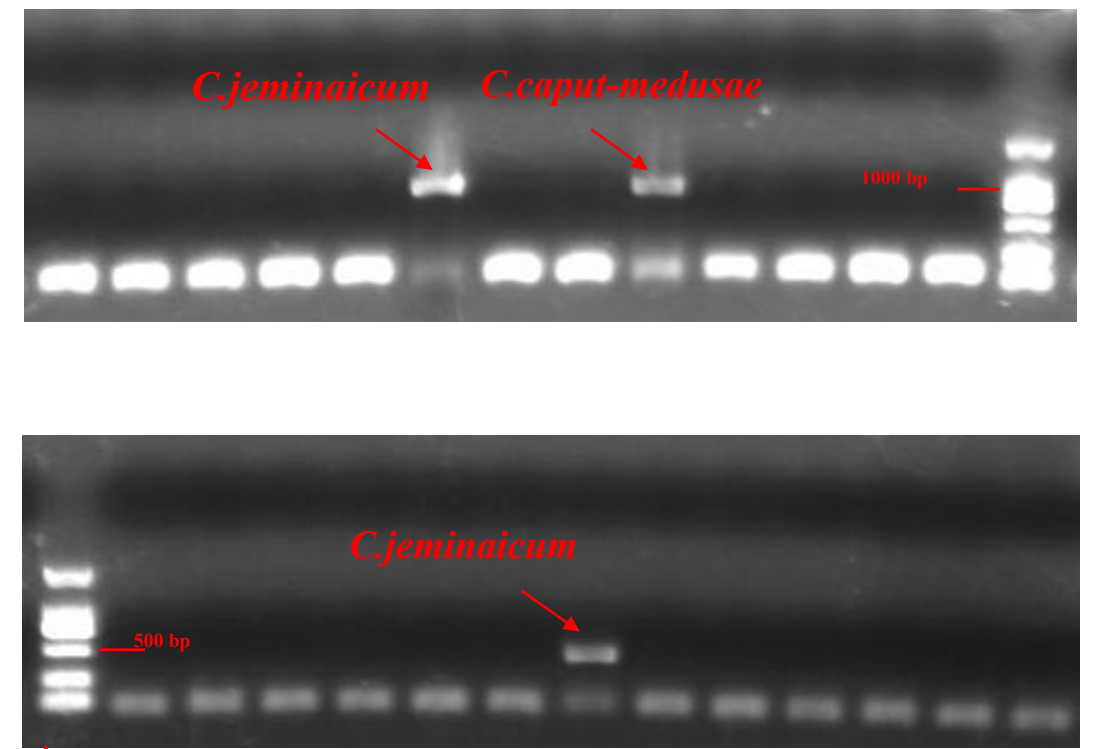

Supplement: Supplementary file 2 — Additional file 2: Figure S1. Sequence identity plots for 21 assembled Calligonum chloroplast. Genomes, with C. jeminaicum as reference (left). Special insertion (or deletion) test results (right). Segments I: about 800 bp, segments II: about 400 bp. [file 12870_2020_2466_MOESM2_ESM.pdf]

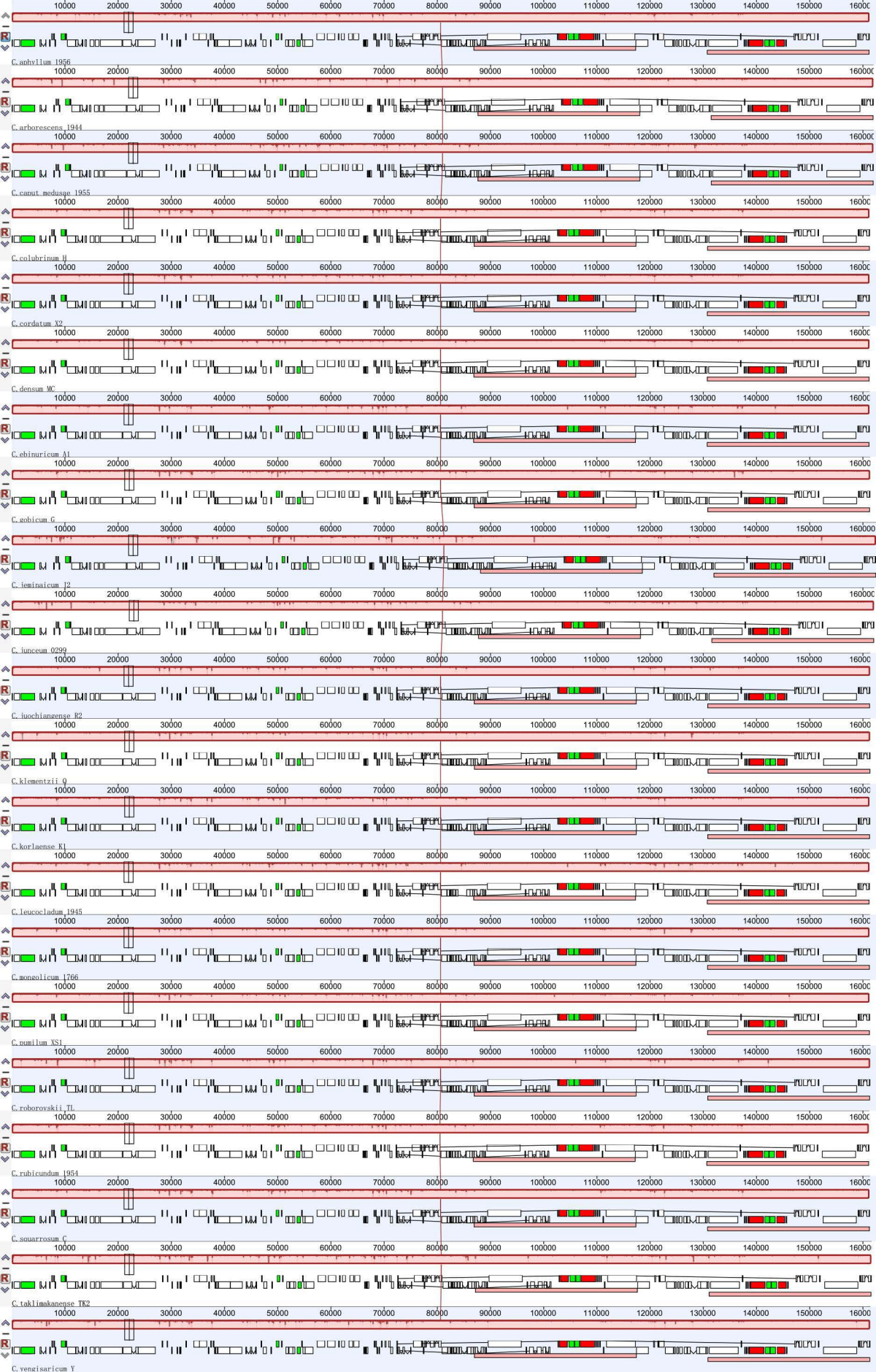

Supplement: Supplementary file 5 — Additional file 5: Figure S3. Genome rearrangement events of 21 assembled Calligonum species. [file 12870_2020_2466_MOESM5_ESM.pdf]

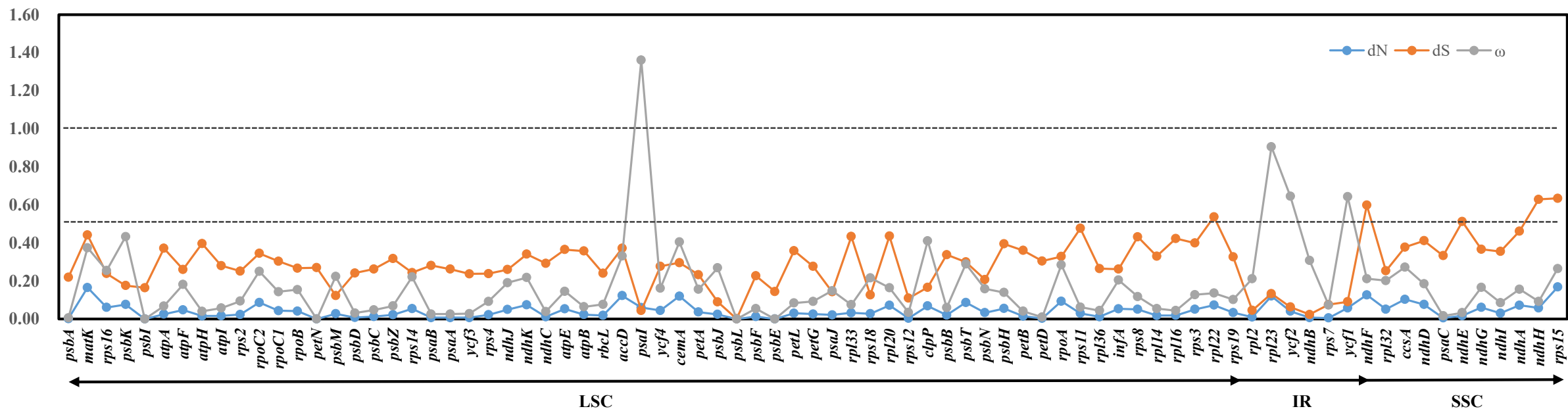

Supplement: Supplementary file 7 — Additional file 7: Figure S4. The dN / dS (ω) value of protein-coding genes from Calligonum plastid genomes. [file 12870_2020_2466_MOESM7_ESM.pdf]

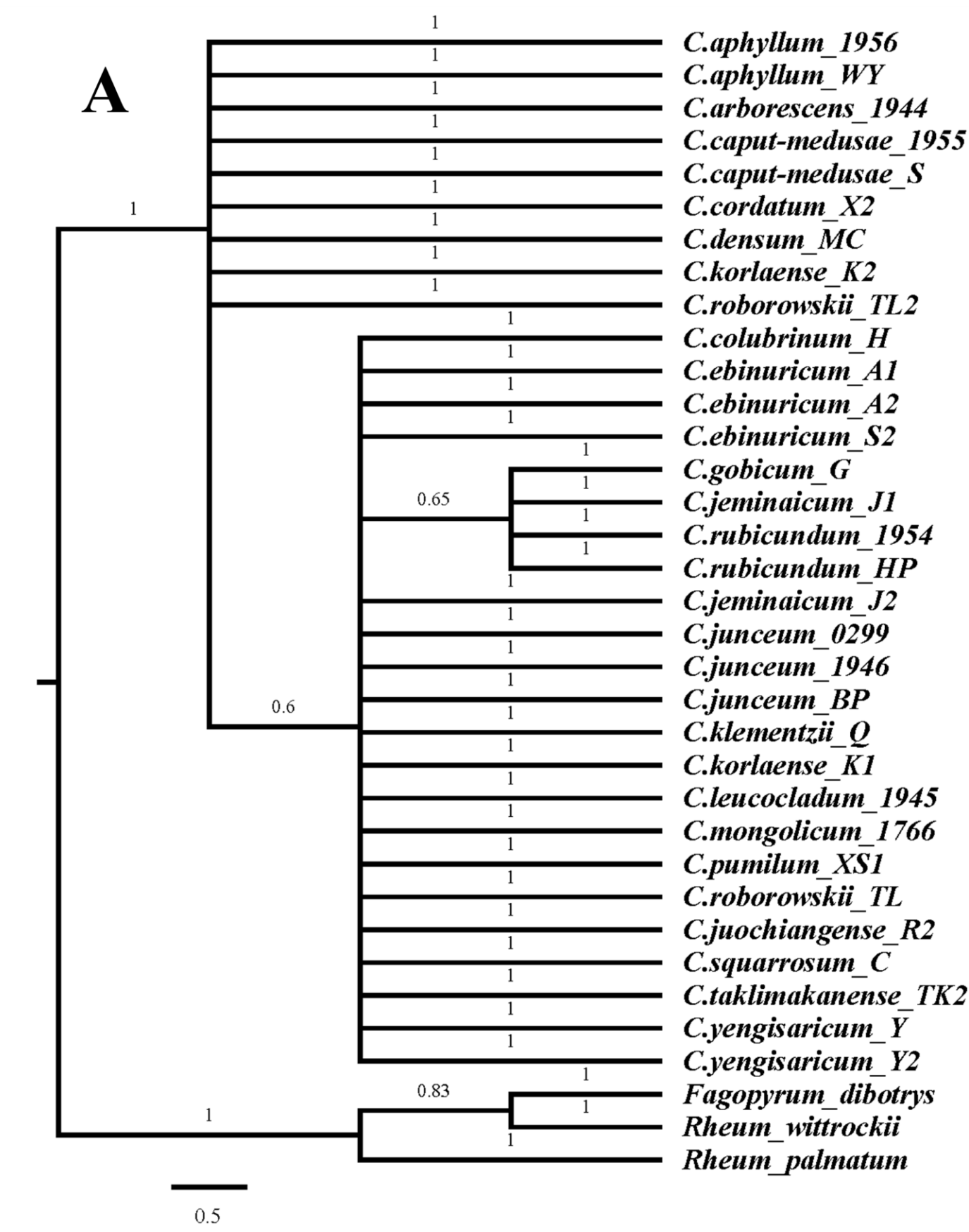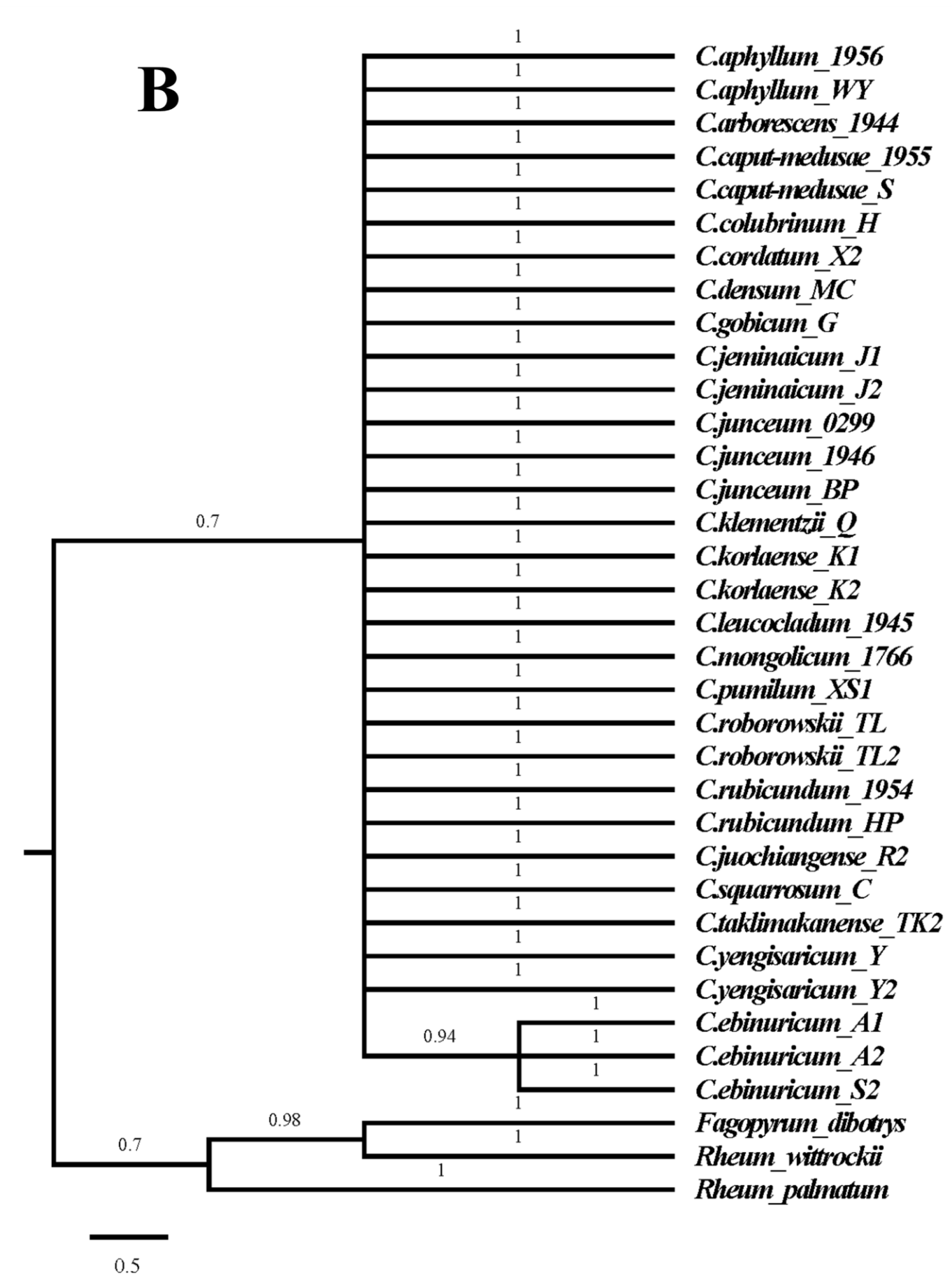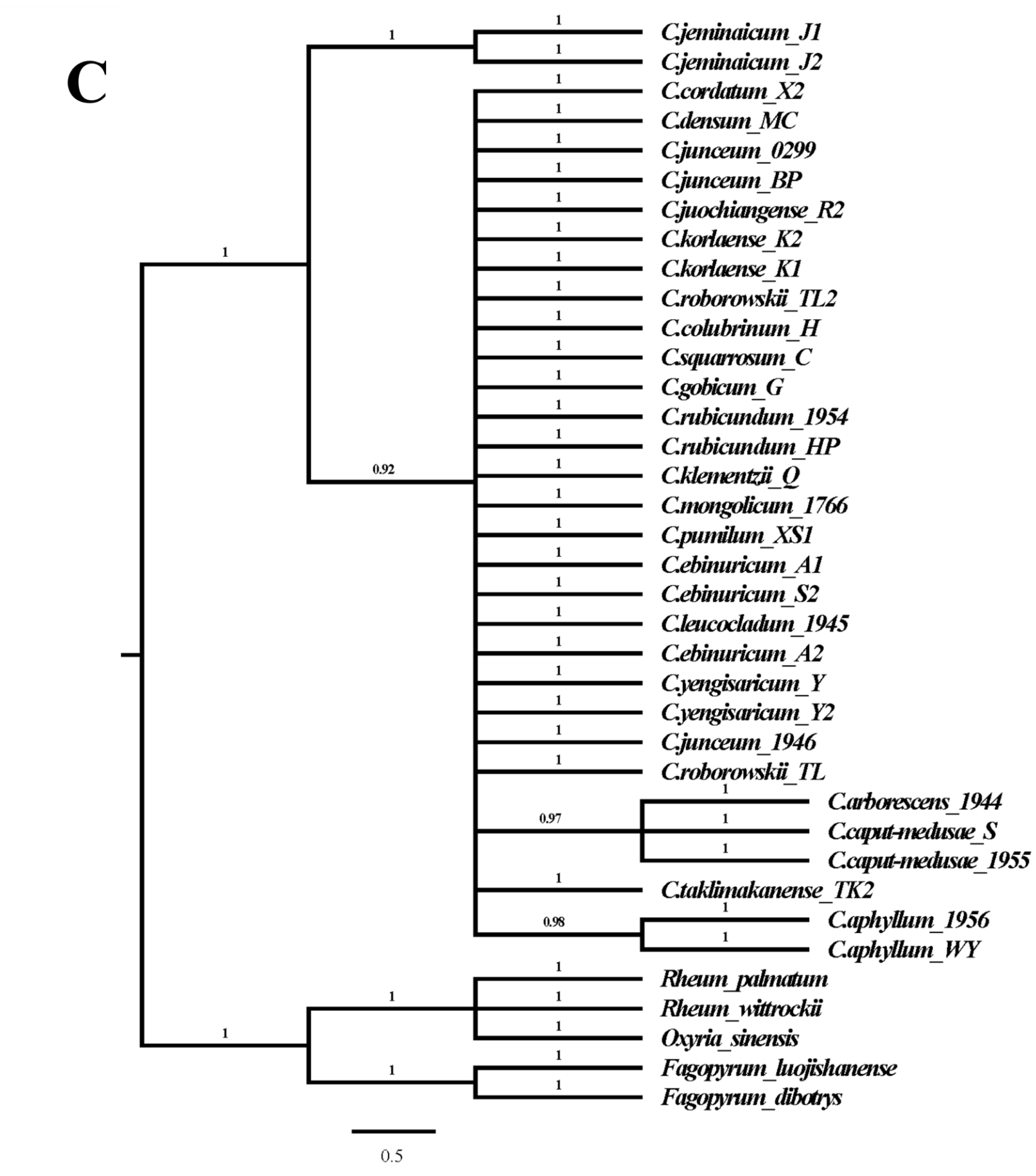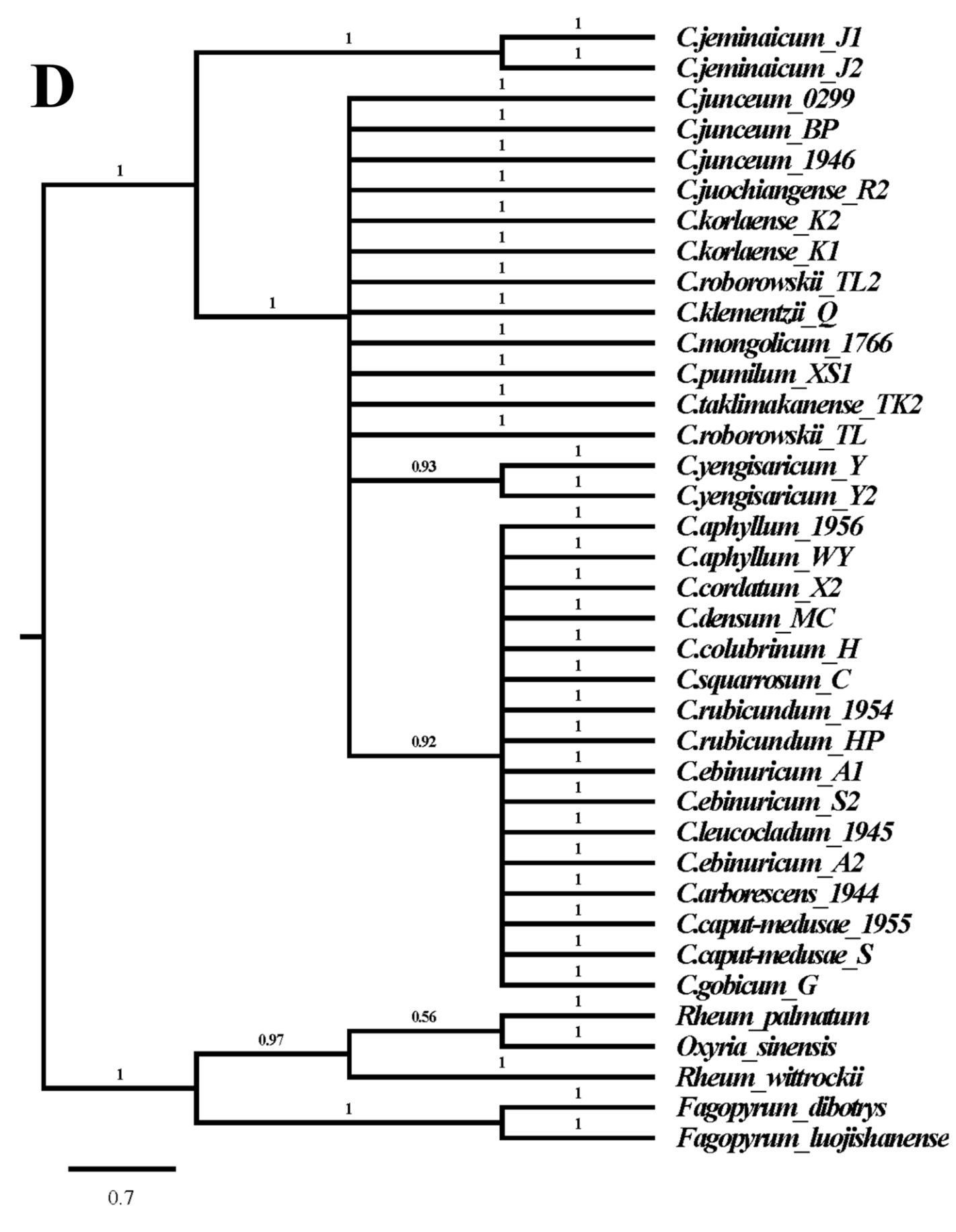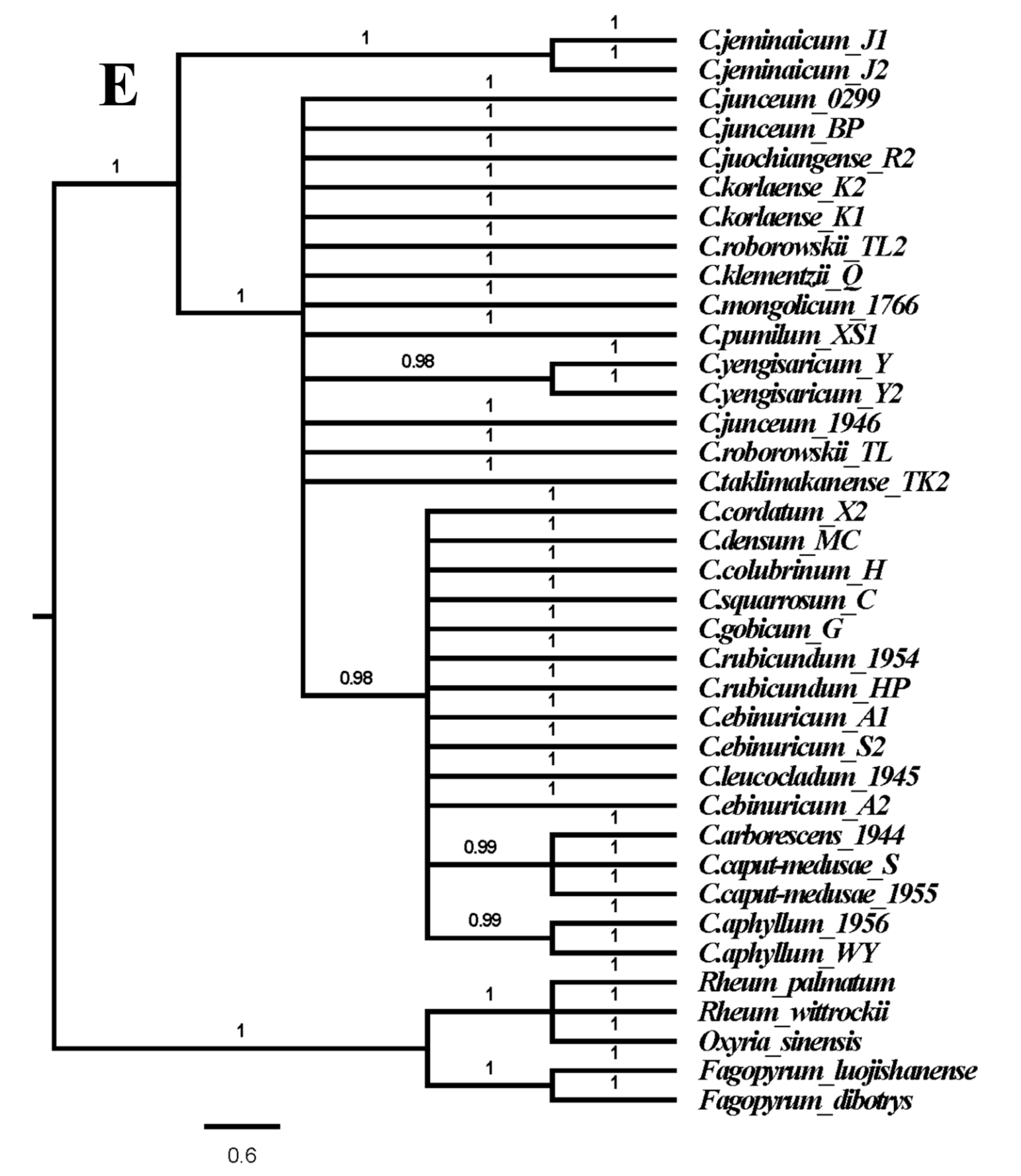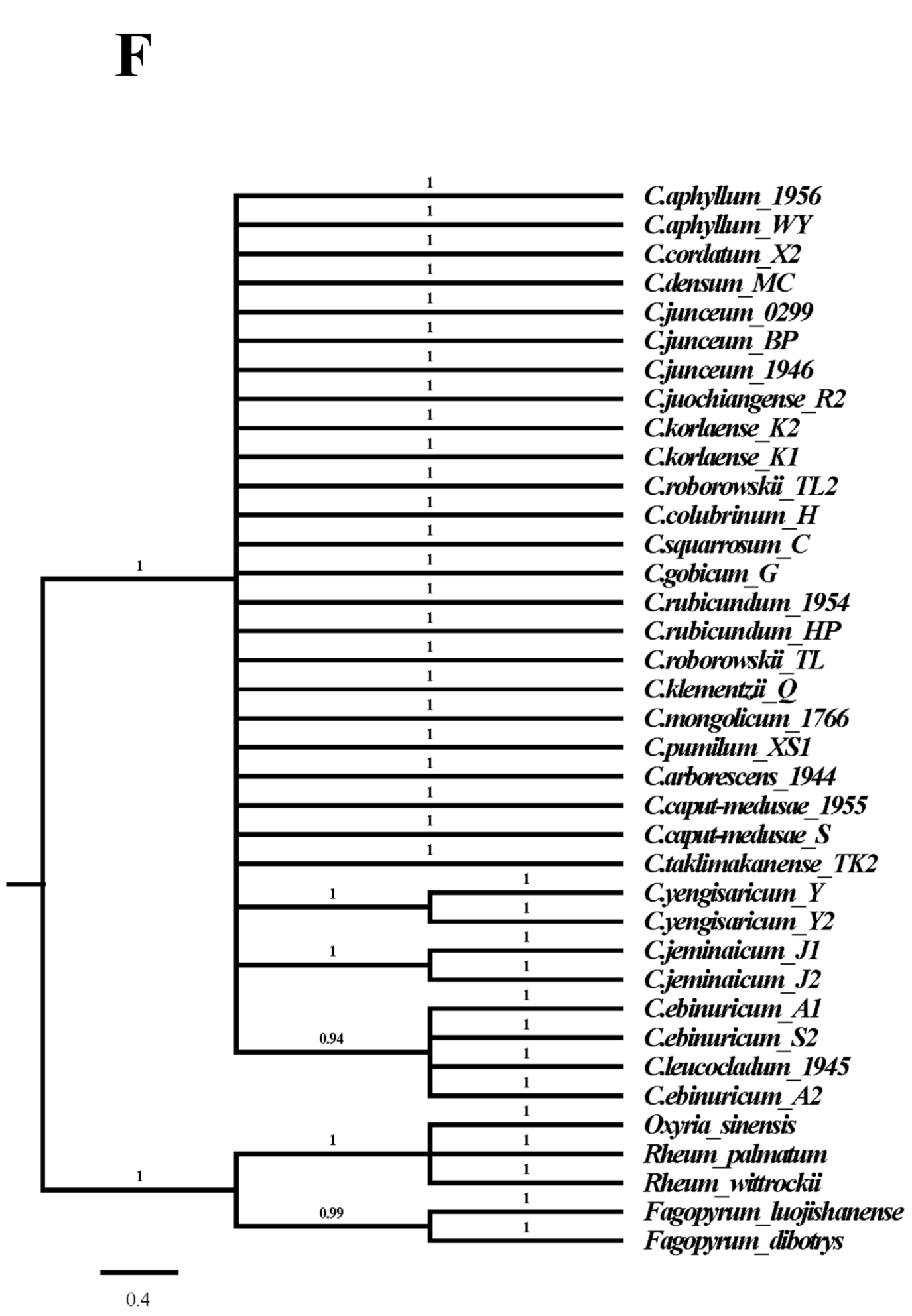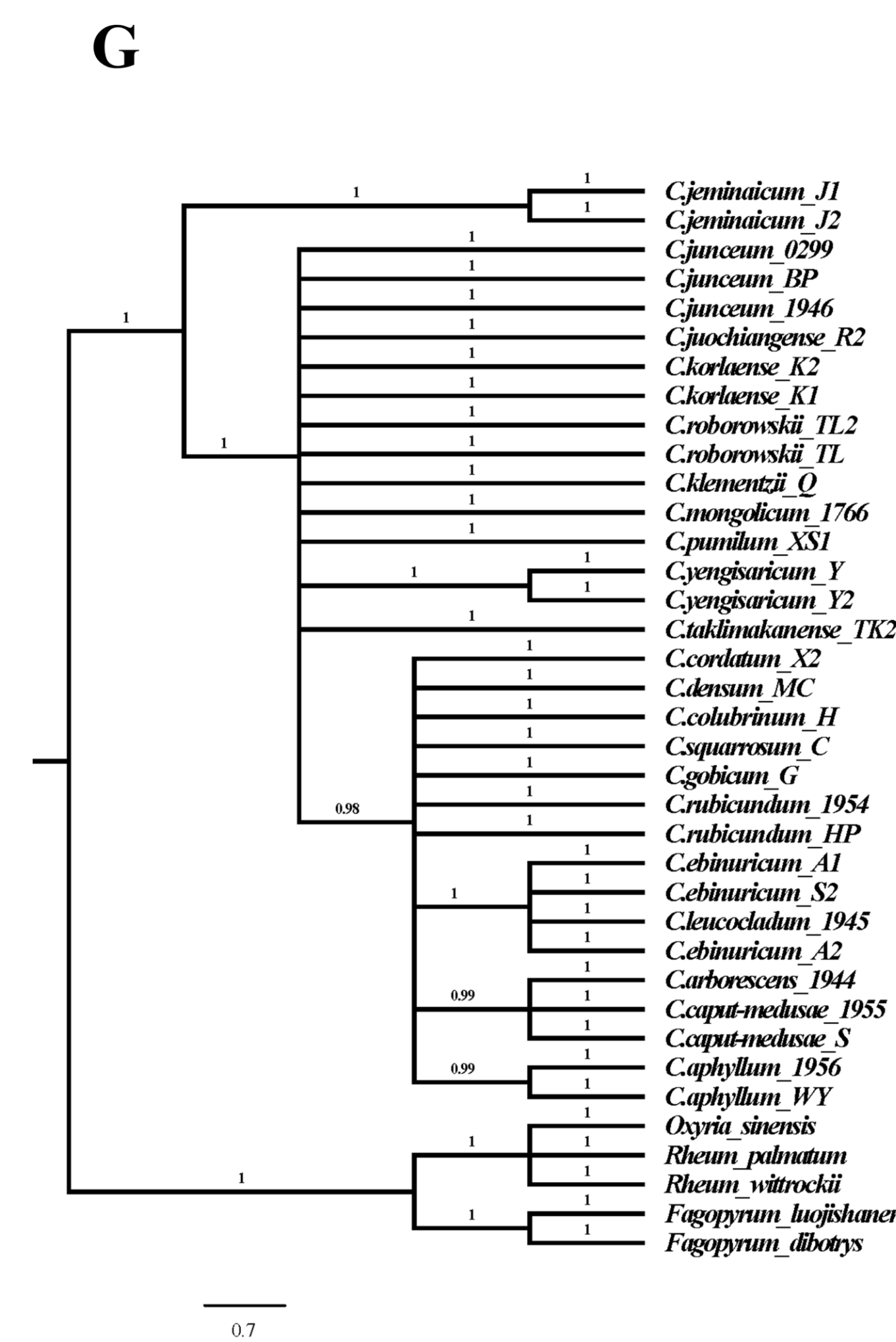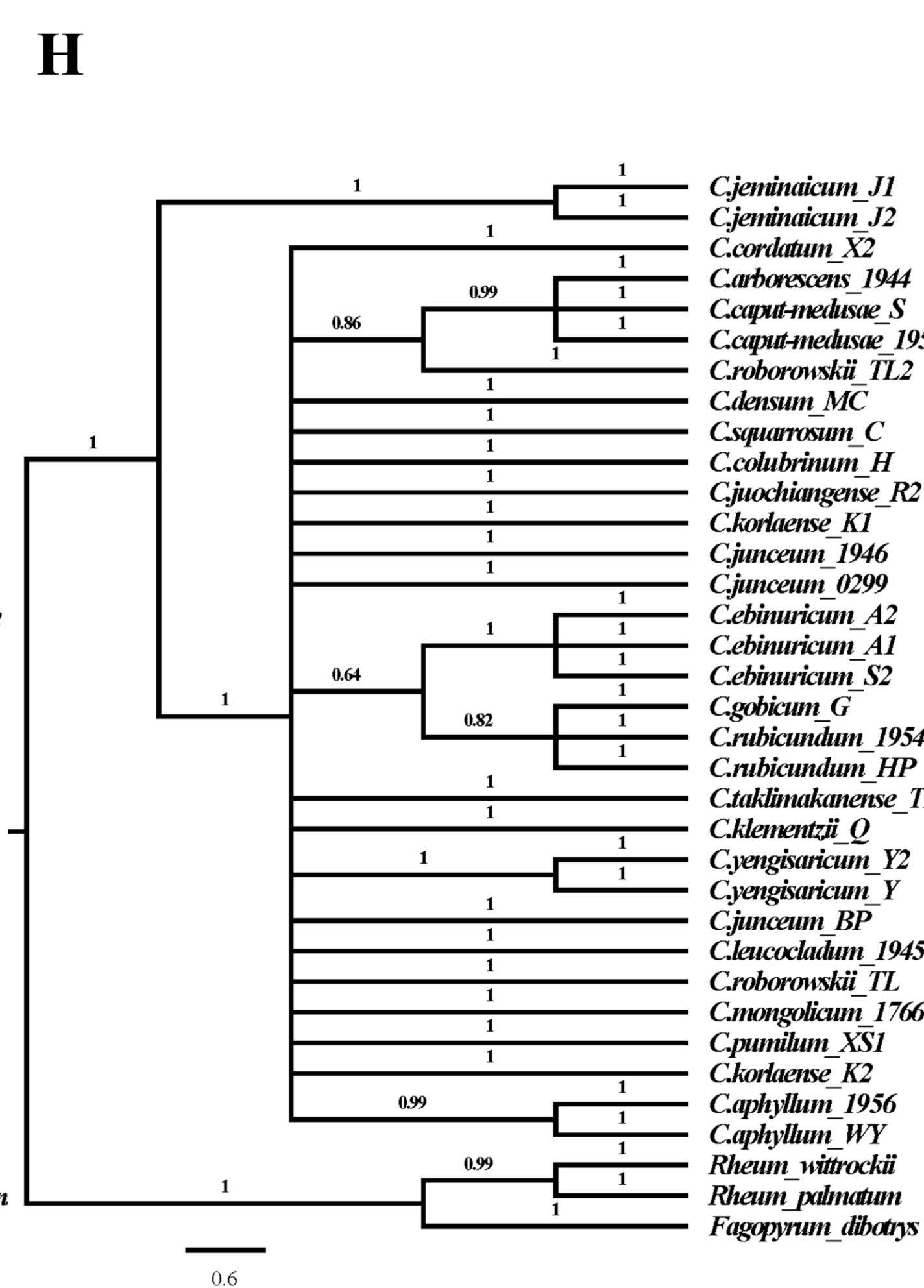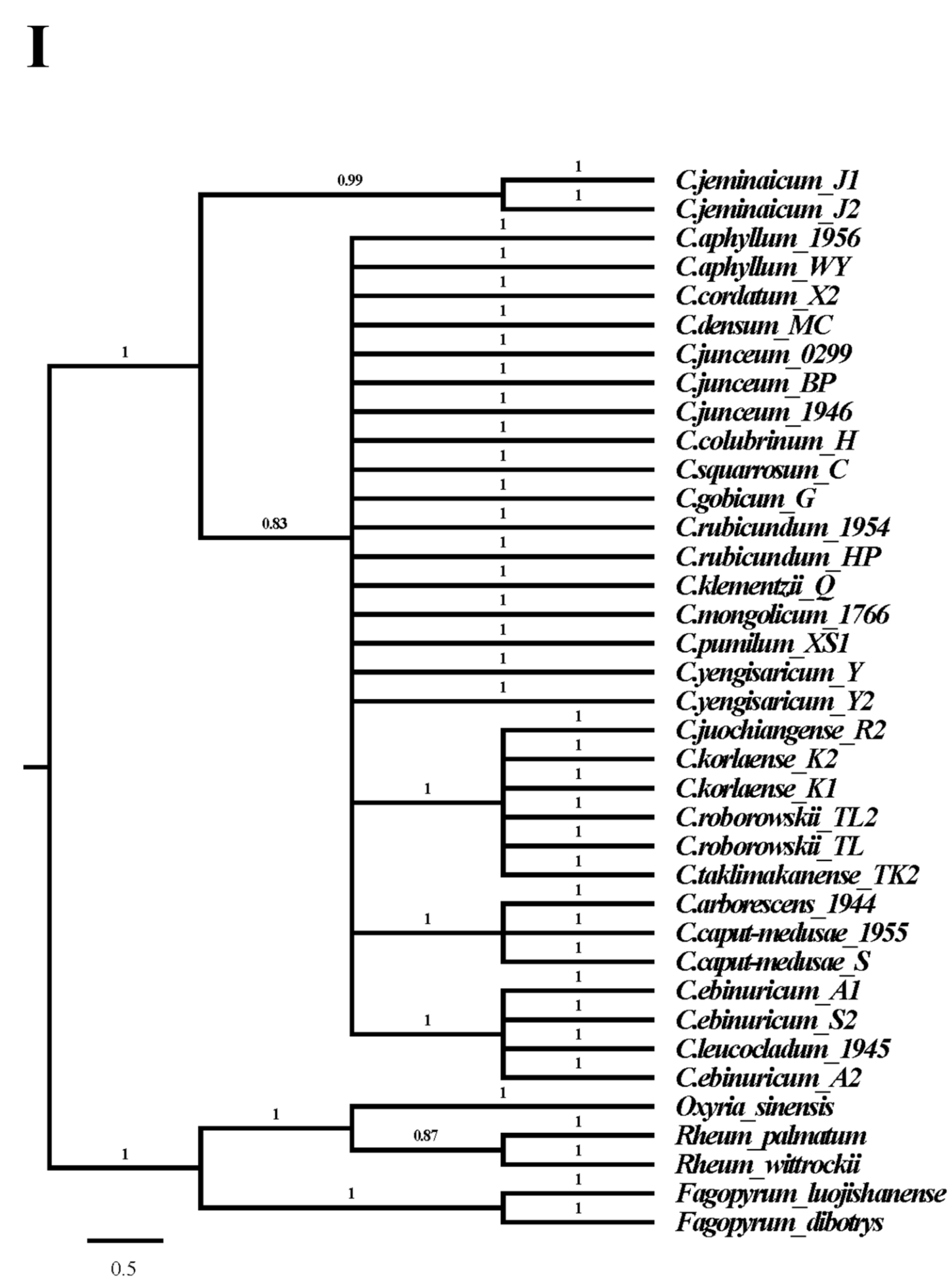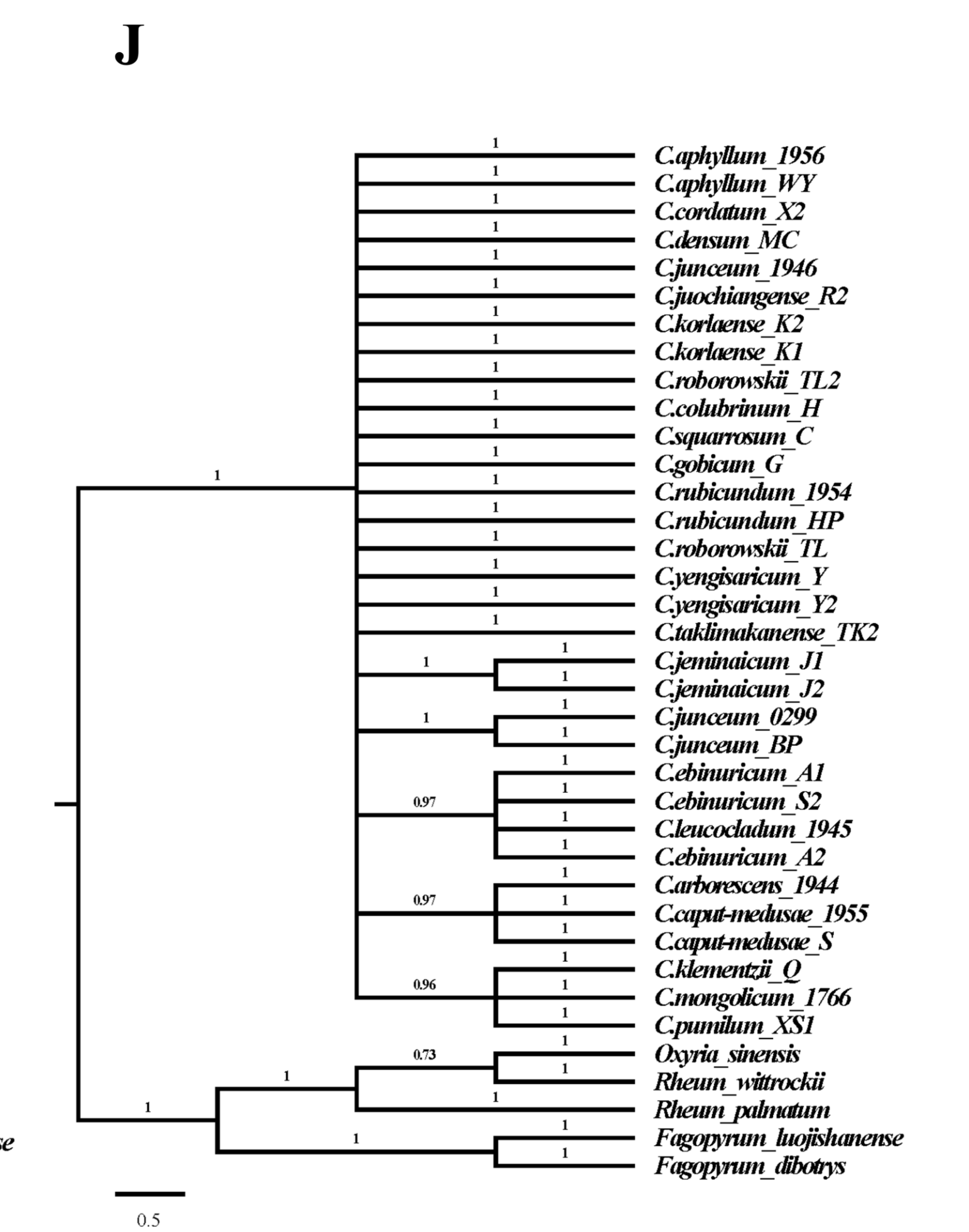

Supplement: Supplementary file 9 — Additional file 9: Figure S6. Bayesian tree inferred from different core-barcodes of the Calligonum. Numbers above branches indicate posterior probabilities (A: ITS1 + 5.8S; B: 5.8S + ITS2; C: matK; D: rbcL E: matK + rbcL; F: trnH-psbA; G: matK + rbcL + trnH-psbA; H: matK + rbcL + trnH-psbA + ITS; I: trnE-T; J: trnT-L). [file 12870_2020_2466_MOESM9_ESM.pdf]
