# Supplementary material for: Complete plastome sequencing resolves taxonomic relationships among species of Calligonum L. (Polygonaceae) in China
Source: BMC Plant Biol. 2020 Jun 8;20:261. doi: 10.1186/s12870-020-02466-5 (PMC7282103; doi:10.1186/s12870-020-02466-5)
Supplement: Supplementary file 3 — Additional file 3: Figure S2. Sequence identity plots for six Polygonaceae genera plastid genomes, with C. jeminaicum as a reference (left). Red boxes represent two special insertion (or deletion) segments I (about 800 bp) and II (about 400 bp). [file 12870_2020_2466_MOESM3_ESM.pdf]

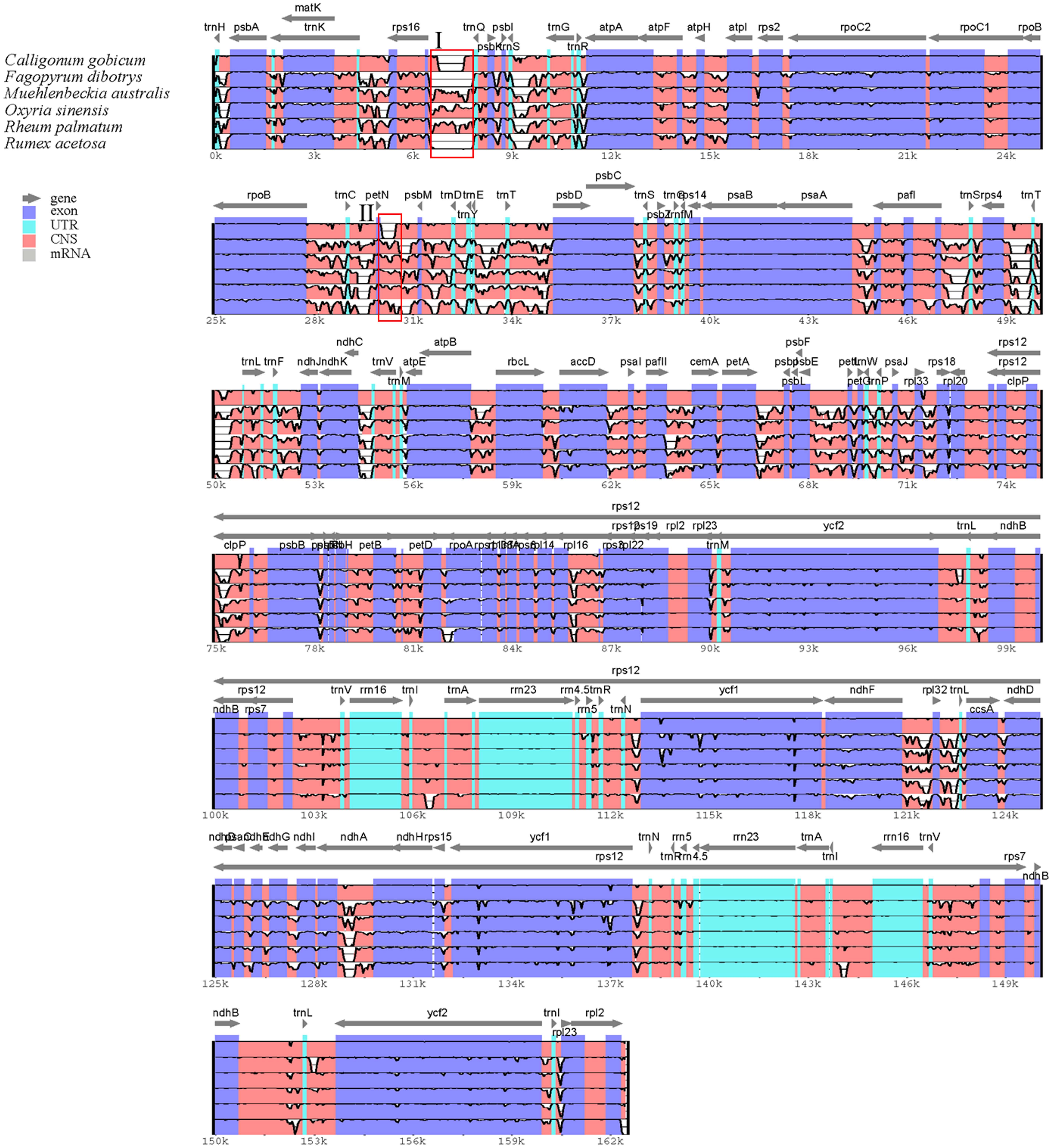
Calligonum gobicum
Fagopyrum dibotrys
Muehlenbeckia australis
Oxyria sinensis
Rheum palmatum
Rumex acetosa
gene
exon
UTR
CNS
mRNA
I
II
trnH psbA matK trnK rps16 trnQ psbI trnG atpA atpF atpH atpI rps2 rpoC2 rpoC1 rpoB
0k 3k 6k 9k 12k 15k 18k 21k 24k
rpoB trnC petN psbM trnD trnE trnT psbC psbD trnS psaB psaA pafI trnT
25k 28k 31k 34k 37k 40k 43k 46k 49k
ndhC atpB trnL trnF trnV atpE rbcL accD psaI pafII cemA petA psbF rps18 rps12 clpP
50k 53k 56k 59k 62k 65k 68k 71k 74k
rps12 clpP psbB petB petD rpl14 rpl16 rpl22 rpl2 rpl23 ycf2 trnL ndhB trnM
75k 78k 81k 84k 87k 90k 93k 96k 99k
rps12 ndhB rps7 trnV rrn16 trnI trnA rrn23 rrn5 trnN ycf1 ndhF rpl32 trnL ccsA ndhD
100k 103k 106k 109k 112k 115k 118k 121k 124k
ndhI ndhA rps15 ycf1 trnN rrn5 rrn23 trnA rrn16 trnV rps12 trnI rps7 ndhB
125k 128k 131k 134k 137k 140k 143k 146k 149k
ndhB trnL ycf2 trnI rpl2 rpl23
150k 153k 156k 159k 162k
