# Supplementary material for: Complete plastome sequencing resolves taxonomic relationships among species of Calligonum L. (Polygonaceae) in China
Source: BMC Plant Biol. 2020 Jun 8;20:261. doi: 10.1186/s12870-020-02466-5 (PMC7282103; doi:10.1186/s12870-020-02466-5)
Supplement: Supplementary file 4 — Additional file 4: Table S2. Primers and samples for special insertion test. [file 12870_2020_2466_MOESM4_ESM.docx]

**Table S2** Primers and samples for special insertion test

| **Location / kb** | **Primer name** | **Sequence** | **Samples** |
| --- | --- | --- | --- |
| 7 – 9 (Ⅰ) | CA-800-F | AGAAATTCGAAATAGAAGGGTAGAT | *C. cordatum*, *C. korlaense*, *C. gobicum*,  *C. klementzii*, *C. roborowskii*,  *C. jeminaicum*, *C. ebinuricum*,  *C. colubrinum*,  *C. squarrosum*, *C. yengisaricum*,  *C. taklimakanense*, *C. juochiangense*,  *C. caput-medusae* |
|  | CA-800-R | CGTTACTTATGCCGCGTGT |  |
| 29 – 31(Ⅱ) | CA-400-F | CGGTAAGGAATCAAACTTTCTCA |  |
|  | CA-400-R | TGAGTCGATGTCGATAAAGCA |  |
