# Supplementary material for: Complete plastome sequencing resolves taxonomic relationships among species of Calligonum L. (Polygonaceae) in China
Source: BMC Plant Biol. 2020 Jun 8;20:261. doi: 10.1186/s12870-020-02466-5 (PMC7282103; doi:10.1186/s12870-020-02466-5)
Supplement: Supplementary file 8 — Additional file 8: Figure S5. Three sections of Calligonum based on the flora of Iran (Rechinger & Schiman-Czeika, 1986). The colors represent different sections. Numbers above branches indicate posterior probabilities (PP, left) and the ML bootstrap values (BS, right). Branches with* have PP = 1 and BS = 100%. [file 12870_2020_2466_MOESM8_ESM.pdf]

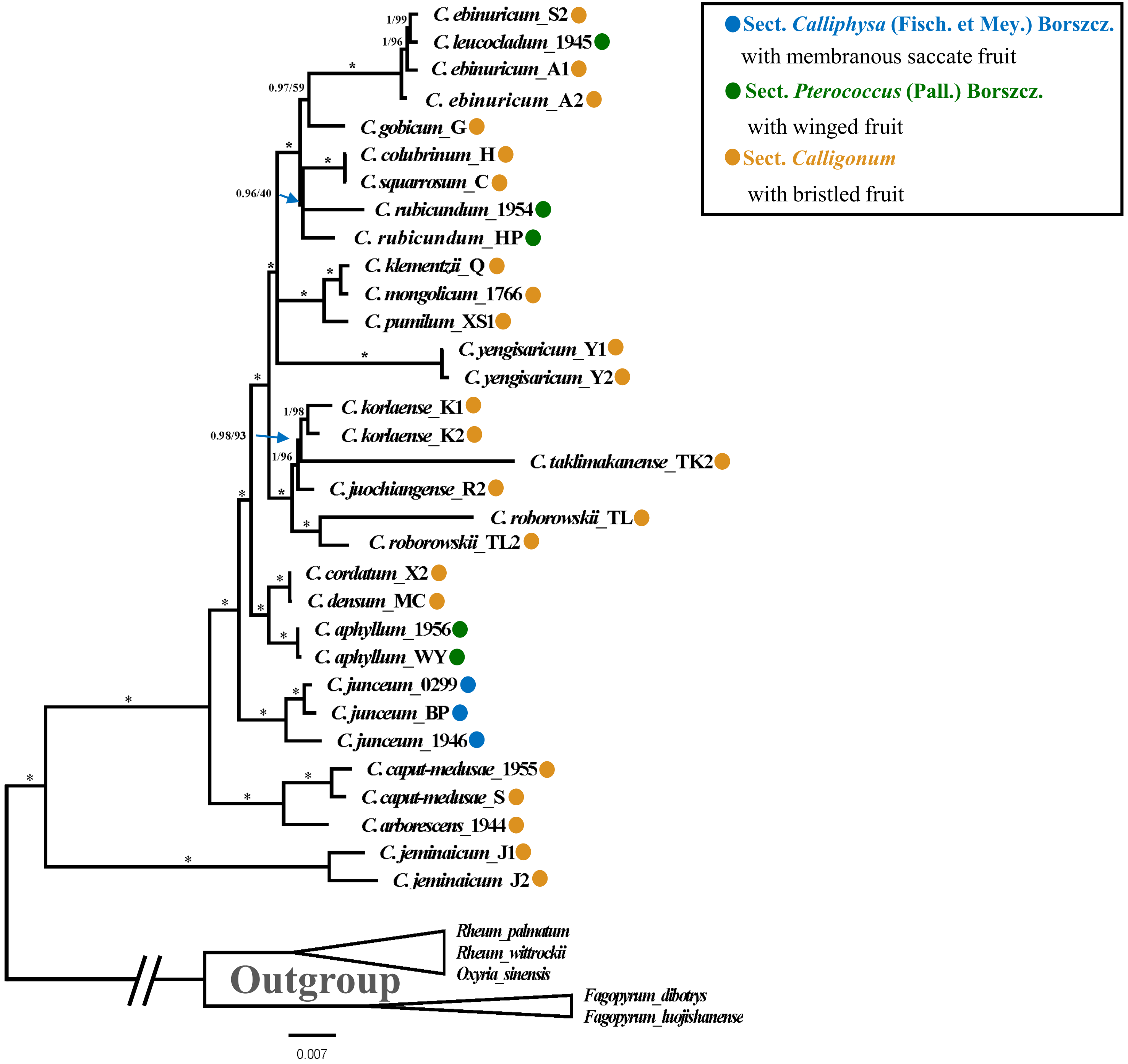
Sect. Calliphysa (Fisch. et Mey.) Borszcz.
with membranous saccate fruit
Sect. Pterococcus (Pall.) Borszcz.
with winged fruit
Sect. Calligonum
with bristled fruit
C. ebinuricum_S2
C. leucocladum_1945
C. ebinuricum_A1
C. ebinuricum_A2
C. gobicum_G
C. colubrinum_H
C. squarrosum_C
C. rubicundum_1954
C. rubicundum_HP
C. klementzii_Q
C. mongolicum_1766
C. pumilum_XS1
C. yengisaricum_Y1
C. yengisaricum_Y2
C. korlaense_K1
C. korlaense_K2
C. taklimakanense_TK2
C. juochiangense_R2
C. roborowskii_TL
C. roborowskii_TL2
C. cordatum_X2
C. densum_MC
C. aphyllum_1956
C. aphyllum_WY
C. junceum_0299
C. junceum_BP
C. junceum_1946
C. caput-medusae_1955
C. caput-medusae_S
C. arborescens_1944
C. jeminaicum_J1
C. jeminaicum_J2
1/99
1/96
0.97/59
0.96/40
1/98
0.98/93
1/96
Outgroup
Rheum_palmatum
Rheum_wittrockii
Oxyria_sinensis
Fagopyrum_dibotrys
Fagopyrum_luojishanense
0.007
