# Supplementary material for: Complete plastome sequencing resolves taxonomic relationships among species of Calligonum L. (Polygonaceae) in China
Source: BMC Plant Biol. 2020 Jun 8;20:261. doi: 10.1186/s12870-020-02466-5 (PMC7282103; doi:10.1186/s12870-020-02466-5)
Supplement: Supplementary file 11 — Additional file 11: Table S3. nrITS sequence assembly information. [file 12870_2020_2466_MOESM11_ESM.docx]

Table S3 nrITS sequence assembly information

| Species | sample-ID | Average coverage | length | heterozygous sites (proportion) |
| --- | --- | --- | --- | --- |
| *C. aphyllum* | HM1956 | 496.75 | 758 | 2 (0.26%) |
| *C. aphyllum* | SS-WY | 2524.19 | 758 | 1 (0.13%) |
| *C. arborescens* | HM1944 | 2995.49 | 759 | 1 (0.13%) |
| *C. caput-medusae* | HM1955 | 890.25 | 758 | 3 (0.40%) |
| *C. caput-medusae* | SS-S | 2218.95 | 758 | 3 (0.40%) |
| *C. colubrinum* | SS-H | 4084.24 | 765 | 2 (0.26%) |
| *C. cordatum* | SS-X2 | 4634.35 | 758 | 0 (0%) |
| *C. densum* | SS-MC | 1186.08 | 758 | 3 (0.40%) |
| *C. ebinuricum* | SS-A1 | 956.25 | 764 | 0 (0%) |
| *C. ebinuricum* | SS-A2 | 7522.78 | 764 | 0 (0%) |
| *C. ebinuricum* | SS-S2 | 500.28 | 764 | 0 (0%) |
| *C. gobicum* | SS-G | 1926.58 | 765 | 1 (0.13%) |
| *C. jeminaicum* | SS-J1 | 978.06 | 764 | 0 (0%) |
| *C. jeminaicum* | SS-J2 | 4542.89 | 764 | 0 (0%) |
| *C. junceum* | HM0299 | 414.55 | 757 | 3 (0.40%) |
| *C. junceum* | HM1946 | 1328.42 | 765 | 4 (0.52%) |
| *C. juochiangense* | SS-R2 | 7457.75 | 764 | 0 (0%) |
| *C. klementzii* | SS-Q | 4396.26 | 765 | 2 (0.26%) |
| *C. korlaense* | SS-K1 | 808.68 | 764 | 3 (0.40%) |
| *C. korlaense* | SS-K2 | 1415.38 | 759 | 0 (0%) |
| *C. leucocladum* | HM1945 | 1569.04 | 757 | 1 (0.13%) |
| *C. mongolicum* | HM1766 | 306.50 | 702 | 0 (0%) |
| *C. pumilum* | SS-XS1 | 1012.54 | 764 | 0 (0%) |
| *C. roborowskii* | SS-TL | 7508.36 | 764 | 0 (0%) |
| *C. roborowskii* | SS-TL2 | 1400.54 | 759 | 0 (0%) |
| *C. rubicundum* | HM1954 | 393.41 | 764 | 1 (0.13%) |
| *C. rubicundum* | SS-HP | 1396.51 | 764 | 1 (0.13%) |
| *C. squarrosum* | SS-C | 4531.57 | 765 | 2 (0.26%) |
| *C. taklimakanense* | SS-TK2 | 3893.54 | 764 | 3 (0.40%) |
| *C. yengisaricum* | SS-Y | 2974.26 | 764 | 0 (0%) |
| *C. yengisaricum* | SS-Y2 | 696.62 | 764 | 0 (0%) |
| *C. junceum* | SS-BP | 4572.44 | 757 | 0 (0%) |
