# Supplementary material for: Complete plastome sequencing resolves taxonomic relationships among species of Calligonum L. (Polygonaceae) in China
Source: BMC Plant Biol. 2020 Jun 8;20:261. doi: 10.1186/s12870-020-02466-5 (PMC7282103; doi:10.1186/s12870-020-02466-5)
Supplement: Supplementary file 12 — Additional file 12: Table S4. Molecular models selected for all the dataset. [file 12870_2020_2466_MOESM12_ESM.docx]

**Table S4** Molecular models selected for all the dataset

| **Data set** | | **Best fit model** | **Model selection** |
| --- | --- | --- | --- |
| Plastid genome | | TVM+I+G | GTR+G+I |
| Standard DNA barcodes | nrITS | TrN+G | GTR+G |
|  | *matK* | TVM+G | GTR+G |
|  | *rbcL* | TPM1uf+G | GTR+G |
|  | *trnH-psbA* | TVM | GTR |
|  | combination | TPM1uf+G | GTR+G |
| Taxon-specific barcodes | *ndhF* | TVM+G | GTR+G |
|  | *ndhF*-*rpl32* | TVM+G | GTR+G |
|  | *rpl32*-*trnL* | TPM1uf+I | GTR+I |
|  | *trnT-L* | TPM1uf+G | GTR+G |
|  | *trnC*-*petN* | TPM1uf+G | GTR+G |
|  | *trnE-T* | TPM1uf+G | GTR+G |
|  | *trnS-G* | TVM+G | GTR+G |
|  | combinations | TPM1uf+G | GTR+G |
